# Possible Peierls Distortion Through Re_2_ Dimer Formation in the LaNiO_2_‐Type Nitrides *Ln*ReN_2_ (*Ln* = Pr, Nd)

**DOI:** 10.1002/anie.202519710

**Published:** 2025-12-31

**Authors:** Dominik Werhahn, Amalina T. Buda, Simon Steinberg, Pascal Manuel, Clemens Ritter, J. Paul Attfield, Simon D. Kloß

**Affiliations:** ^1^ Department Chemistry LMU Munich Butenandtstr. 5–13 81377 Munich Germany; ^2^ Institute of Inorganic Chemistry RWTH Aachen University Landoltweg 1 52074 Aachen Germany; ^3^ ISIS Neutron and Muon Source STFC Rutherford Appleton Laboratory Didcot UK; ^4^ Institut Laue‐Langevin 71 avenue des Martyrs, CS 20156, 38042 Grenoble Cedex 9 France; ^5^ Centre for Science at Extreme Conditions University of Edinburgh Peter Guthrie Tait Road, EH9 3FD Edinburgh UK

**Keywords:** High‐pressure chemistry, Magnetic properties, Metal−metal interactions, Neutron diffraction, Solid‐state structures

## Abstract

Nitride perovskites are a recently opened class of materials with a limited number of stable compounds. Notably, anion‐vacancy‐ordered materials such as LaNiO_2_‐type nitrides are rare despite useful properties such as superconductivity reported for analogous oxides. Here, we report the preparation of two *Ln*ReN_2_ (*Ln* = Pr, Nd) materials with a distorted LaNiO_2_‐type structure at high‐pressure, high‐temperature conditions of a large volume press. Powder X‐ray and neutron diffraction show that the orthorhombic distortion (o‐*Ln*ReN_2_) of the LaNiO_2_‐aristotype structure is caused by buckling of the ReN_4/2_ layers through Re dimerization. Electronic structure analysis reveals that the o‐*Ln*ReN_2_ materials are composed of classic nitridometallate anions and a cationic intermetallic framework. Moreover, the calculations reveal the driving force of the Re dimerization likely to be from an electronic instability related to a Peierls‐type distortion. We further characterize the magnetic ground state of both materials with magnetization measurements as well as magnetic neutron scattering uncovering a long‐range antiferromagnetic order of Nd^3+^ moments in NdReN_2_ at T_N_ = 15.5 K. The stability of the compounds is investigated by temperature‐dependent powder X‐ray diffraction showing decomposition of NdReN_2_ at ca. 750 °C. The o‐*Ln*ReN_2_ materials represent a curious class of nitrides at the border of classic nitridometallates, intermetallics, and low‐dimensional complex chemistry.

## Graphical abstract

This study presents the high‐pressure synthesis of two new anion‐vacancy‐ordered perovskite‐related nitrides, PrReN_2_ and NdReN_2_, featuring distorted LaNiO_2_‐type structures. X‐ray and neutron diffraction reveal a structural distortion driven by dimerization of Re‐chains, while electronic structure calculations reveal the simultaneous presence of nitridometallate anions and an intermetallic framework. Magnetic properties were studied with neutron diffraction and magnetization measurements, which reveal an antiferromagnetic ordering of Nd^3+^ moments in NdReN_2_ at 15.5 K.

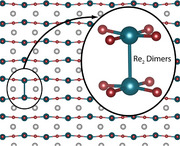

## Introduction

Perovskite and perovskite‐related nitrides represent a rapidly expanding materials family emerging through high‐pressure (HP) synthesis of LaReN_3_ and physical vapor deposition (PVD) preparation of LaWN_3_.^[^ ^1^, ^2^ ^]^ This class now includes further ABN_3_‐type compounds such as CeMoN_3_, CeWN_3_, CeTaN_3_ (PVD/HP), GdWN_3_ (PVD), the Ruddlesden–Popper nitrides Ce_2_TaN_4_ and *Ln*_2_ReN_4_ with *Ln* = Pr, Nd (HP), and molecular antiperovskites La_3_ *M*N_5_ with *M* = Cr, Mn, Mo (HP).^[^ ^3^, ^4^, ^5^, ^6^, ^7^, ^8^ ^]^ The extended framework structures of perovskite types in general lead to numerous cooperative physical properties and exotic bonding characteristics were already observed in nitrides such as orbital ordering in LaReN_3_ through a combination of Jahn–Teller and spin‐orbit coupling, while a second order Jahn–Teller effect led to piezoelectricity in LaWN_3_ and ferroelectricity in CeTaN_3_.^[^ ^1^, ^2^, ^5^, ^9^, ^10^ ^]^

Beyond stoichiometric perovskite‐type phases, reduced, anion‐vacancy‐ordered derivatives are also important as highlighted by infinite layer LaNiO_2_‐types following the discovery of unconventional superconductivity in Sr‐doped NdNiO_2_ as well as bulk transitions in hole‐doped SmNiO_2_ (*T*_c_ = 40 K) and Ca‐doped LaNiO_2_, and also spin glass behavior in bulk‐phase LaNiO_2_‐types.^[^ ^11^, ^12^, ^13^, ^14^ ^]^ In stark contrast, anion‐vacancy‐ordered nitride analogues are almost unexplored. Only one LaNiO_2_‐type nitride was prepared through topotactic decomposition of LaReN_3_ to LaReN_2_, which resulted in a poorly crystalline powder.^[^ ^15^ ^]^ Anion‐vacancy ordered Ruddlesden–Popper nitrides A_1+n_B_n_N_3n_ with *n* = 1 and 2 such as Ce_2_ *M*N_3_ (*M* = Cr, Mn) or La_3_ *M*_2_N_6_ (*M* = V, Cr) are known from ambient pressure synthesis.^[^ ^16^, ^17^, ^18^, ^19^ ^]^ Most notably, topotactic decomposition of *Ln*_2_ReN_4_ (*Ln* = Pr, Nd) phases gave new *Ln*_2_ReN_3_ materials in which disordered Re displacements consistent with formation of short and long Re– distances, i.e., unbridged Re_2_ dimers, were observed.^[^ ^3^ ^]^

Such metal–metal interactions in solid‐state materials can lead to interesting phenomena like superconductivity in doped Mott insulators such as the cuprates, the Verwey transition in Fe_3_O_4_ with trimeron formation, the metal–insulator transition in VO_2_, and charge ordering in reduced niobium oxyfluoride Nb_2_O_2_F_3_.^[^ ^20^, ^21^, ^22^, ^23^ ^]^ Materials classes where direct metal–metal bonding is observed are, for example, low‐valent main group and early transition metals ranging from alkali metal suboxides to reduced rare‐earth halides and transition metal halide/chalcogenide clusters, the latter being famous for superconducting Chevrel phases such as PbMo_6_S_8_.^[^ ^24^, ^25^ ^]^ In nitrides, direct metal–metal interactions are rare but [*M*N_3_]_2_ dimers have been reported for nitridomanganates Li_6_ *AE*_2_[Mn_2_N_6_] (*AE* = Ca, Sr), and the reduced nitridotetrelates *AE*Si_6_N_8_ (*AE* = Sr, Ba) and *AE*_6_Ge_2_N_6_ (*AE* = Ca, Sr).^[^ ^26^, ^27^, ^28^, ^29^, ^30^, ^31^ ^]^ These dimers are analogous to the famous octachloridodirhenate(III) Re_2_Cl_8_^2−^ anions with quadruple Re– bonding. There is also great interest in materials where uniform metal chains can distort to produce varying *M*– distances (in the simplest case, short bonded and long non‐bonded contacts) in accordance with Peierls theorem. Extended unbridged metal– bonding is known in the complex Krogmann's salts with 1D band dispersion like K_2_[Pt(CN)_4_]Cl_0.32_·2.6H_2_O.^[^ ^32^ ^]^ Krogmann's salts also show interesting physical properties like the electron‐phonon coupling in K_2_[Pt(CN)_4_]Br_0.3_·3H_2_O that manifests as a Kohn–Peierls anomaly.^[^ ^33^, ^34^ ^]^

Here, we present the preparation and properties of orthorhombically distorted LaNiO_2_‐type nitrides o‐*Ln*ReN_2_ (*Ln* = Pr, Nd) via high‐pressure high‐temperature conditions in a multi‐anvil large‐volume‐press. Powder neutron diffraction indicates that the distortion originates from dimerization of Re chains and subsequent layer buckling. The electronic structures of the o‐LnReN_2_ compounds are investigated with DFT‐based calculations that reveal the driving force of the Re dimerization being an energy gain similar to a Peierls‐type distortion, reminiscent of Krogmann's salts. The structure and magnetic properties of the compounds are determined with energy‐dispersive X‐ray spectroscopy, magnetization measurements, as well as powder neutron and X‐ray diffraction.

## Results and Discussion

### Synthesis and Characterization

Both *Ln*ReN_2_ (*Ln* = Pr, Nd) materials were prepared according to Equation 1 under high‐pressure, high‐temperature conditions of 8 GPa and ca. 1000 °C provided by a multi‐anvil large‐volume press (details in the Experimental Section of the Supporting Information). Ammonium azide was used as nitrogen source, as opposed to sodium azide, NaN_3_, used for preparation of LaRe^VI^N_3_, due to the reducing atmosphere created by the presence of molecular hydrogen, which prevents oxidation of Re into higher states.^[^ ^1^ ^]^

(1) $$LnN+\text{Re}+{\text{NH}}_{4}N_{3}\to {\text{LnReN}}_{2}+2H_{2}+3/2N_{2}$$

**Figure 1:**
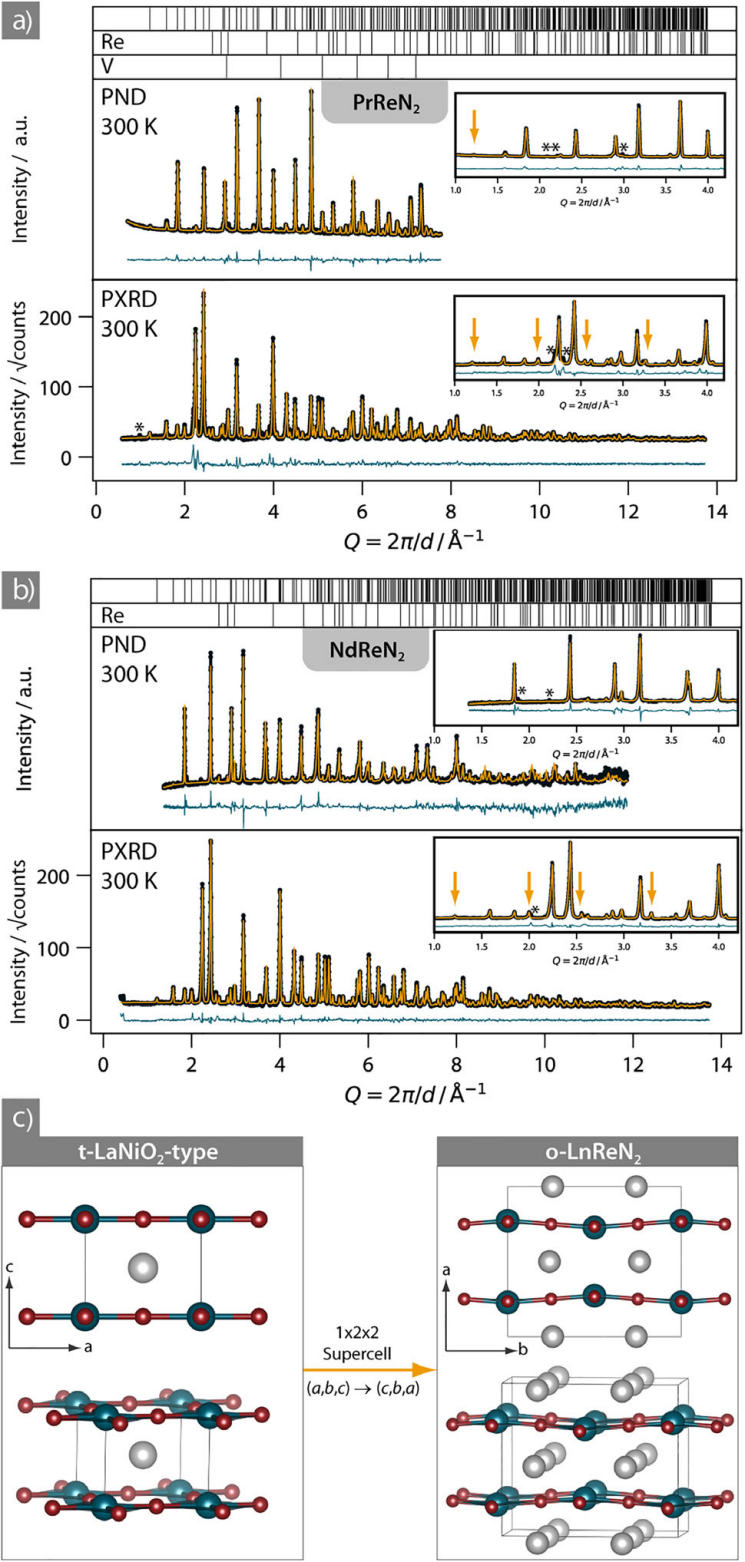
Co‐refinements on powder X‐ray (in‐house) and neutron data collected on a) PrReN_2_ at the D20 beamline at the ILL and b) NdReN_2_ at the WISH beamline at the ISIS Neutron and Muon Source (detector Bank 5). The insets show magnified areas between *Q* = 1 and 4.2 Å^−1^ revealing the superstructure reflections stemming from the orthorhombic distortion. Some superstructure reflection positions are highlighted by arrows, showing the better resolution of X‐ray data for the superstructure. Impurity reflections of unknown byproducts are marked by asterisks. Data are shown as black circles, Rietveld refinement as orange line, difference curve as blue line, theoretical Bragg positions as vertical lines. Vanadium in (a) is from the sample holder (only in PND) while Re is an impurity in (a) and (b). c) Structural relation between the t‐LaNiO_2_ aristotype (left) and the orthorhombic distortion variant (right) of o‐*Ln*ReN_2_. The LaNiO_2_‐type transformation to the supercell is indicated. Right: Two views of the o‐*Ln*ReN_2_ superstructure highlight the MX_4/2_ layer corrugation. M atoms in blue, X atoms in red, and *Ln* atoms in grey.

The samples were obtained as black, microcrystalline powders with metallic luster (Figure S1). Powder diffraction (Figure 1) shows small byproducts of Re‐metal (ca. 5 wt%) in some samples, probably due to diffusion of rare‐earth elements. The elemental composition of the samples was characterized with energy‐dispersive X‐ray spectroscopy, which suggested that both materials are stoichiometric with Pr_0.98(1)_Re_1.0(1)_N_2.0(4)_ and Nd_1.1(1)_Re_1.0(1)_N_2.6(2)_ (calculated excluding 20% oxygen detection). While the Pr compound is air‐stable, the Nd compound was more sensitive to air as indicated by increased detection of oxygen (data in Table S1). Further tentative refinement of nitrogen occupancies on powder neutron diffraction data confirms full nitridation without O substitution (for PrReN_2_ *N*_occ_ = 0.99(1) and for NdReN_2_ at 300 K *N*_occ_ = 0.99(1)).

### Structure Discussion

Powder X‐ray and neutron diffraction data reveal that both *Ln*ReN_2_ (*Ln* = Pr, Nd) materials crystallize in an orthorhombic distortion variant of the tetragonal LaNiO_2_‐type (*P*4/*mmm* (no. 123) → *Cmmm* (no. 65), interchanging *Ammm* (*a*,*b*,*c*) → *Cmmm* (*c*,*b*,*a*)).^[^ ^35^ ^]^ The orthorhombic distortion manifests in superstructure reflections, which are best visible in the powder X‐ray diffraction pattern (Figure 1) as the orthorhombic distortion stems from ordered dislocation of Re‐positions. The superstructure reflections were also observed in the powder neutron diffraction data (PrReN_2_ at D20 of the ILL, NdReN_2_ at WISH of ISIS) but are weaker. Therefore, the best structure models were derived based on a co‐refinement of neutron and X‐ray data at 300 K, to make use of the high sensitivity of neutrons toward nitrogen and the good sensitivity of X‐ray for heavy atoms and lattice parameters (Figure 1a,b). The lattice parameters obtained at 300 K for PrReN_2_ were *a* = 6.85813(11), *b* = 7.9293(6), and *c* = 3.9652(3) Å and for NdReN_2_ *a* = 6.80455(12), *b* = 7.9179(4), and *c* = 3.9595(2) Å). Low‐temperature models of NdReN_2_ were obtained from refinement of long powder neutron diffraction scans at 25 and 1.5 K (Figure S2), while the evolution of lattice parameters was also tracked with a series of short scans (Figure S3). The evolution of lattice parameters shows typical behavior, converging toward a minimum cell of around *V*_min_ = 212.01(4) Å. All positional parameters as well as isotropic displacement parameters (N‐positions were constrained) could be refined and crystallographic information, atomic positions, and a list of interatomic distances and angles are provided in Tables S2–S6. As *Ln*ReN_2_ (*Ln* = Pr, Nd) crystallizes in the orthorhombic distortion variant, the powder diffraction dataset of LaReN_2_, which was previously published with a tetragonal t‐LaNiO_2_‐type model,^[^ ^1^ ^]^ was reinvestigated. The reinvestigation revealed a Re distortion but otherwise remained inconclusive toward a simultaneous orthorhombic metric distortion (details in Supporting Information).

The following structure discussion on the o‐*Ln*ReN_2_ (*Ln* = Pr, Nd) materials is based on data obtained from co‐refinement at 300 K. The t‐LaNiO_2_‐type consists of infinite MX_4/2_ layers, which in o‐*Ln*ReN_2_ buckle owing to an ordered Re displacement perpendicular to the layers, which creates a 1 × 2 × 2 orthorhombic superstructure (Figure 1c). This type of layer buckling has not been reported in infinite layer oxides, so o‐*Ln*ReN_2_ represents a new structure type.^[^ ^36^ ^]^ The Re–N distances in the layers are almost uniform (Pr: *d* = 1.9832(2) and 1.9883(2) Å; Nd: *d* = 1.9804(2) and 1.9844(1) Å) and the layers can therefore be seen as extended [ReN_4/2_]^3−^ nitridometallate anions. The Re displacement, however, leads to long and short Re–Re contacts between layers (Pr: *d*_short_ = 3.122(2) Å and *d*_long_ = 3.736(2) Å, Nd: *d*_short_ = 3.171(7)d Å, and *d*_long_ = 3.605(7) Å). The short contacts are longer than interatomic distances found in rhenium metal (*d* = 2.73 Å), but comparable to those in the dinuclear complex Re_2_(CO)_10_ (*d* = 3.04 Å).^[^ ^37^, ^38^ ^]^ Metal–metal bonded Re dimers with *d*_Re–Re_ ≈ 2.65 Å were reported in Sr_2_CuO_3_‐type nitrides *Ln*_2_ReN_3_ (*Ln* = Pr, Nd) but there, the Re displacements within the ReN_2_N_2/2_ chains were disordered.^[^ ^3^ ^]^ Moreover, in o‐*Ln*ReN_2_ the interlayer *Ln*–*Ln* distances (Pr: *d* = 3.4336(3) Å; Nd: = 3.4062(2) Å; Figure 4c) are shorter than distances in the respective *α*‐La‐type metals (*d*_Pr–Pr_ = 3.67 Å, *d*_Nd–Nd_ = 3.62 Å).^[^ ^39^ ^]^ The overall small metal–metal distances indicate a high degree of metallicity in the o‐*Ln*ReN_2_ materials, which is surprising for a nominal nitridometallate with an extended anion structure. The Re–Re dimers are reminiscent of the octachloridodirhenate [Re_2_Cl_8_]^2−^ anions and their formation may be driven by covalent σ‐ and π‐bonds.^[^ ^31^ ^]^ The simultaneous presence of short *Ln*–*Ln* contacts, however, suggests the existance of an extended cationic intermetallic framework as known from metal‐rich subnitridometallates like Ba_23_Na_11_(*M*N_4_)_4_ with *M* = V, Nb, and Ta and inverse nitride perovskites A_3_BN.^[^ ^40^, ^41^ ^]^ The intricate intergrowth of 2D extended nitridometallate anions and a 3D cationic intermetallic framework is puzzling, which is why the underlying bonding is analyzed with density functional theory (DFT) calculations in a later section.

### Magnetic Properties

#### PrReN_2_

**Figure 2:**
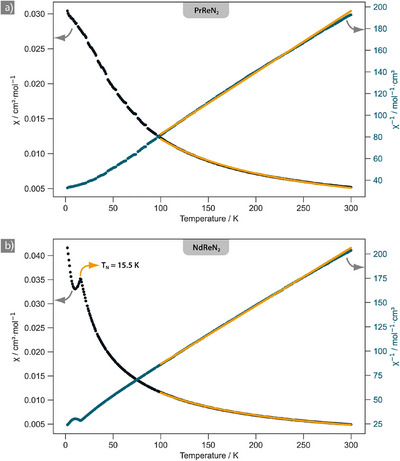
Susceptibility measurement on a sample of a) PrReN_2_ and b) NdReN_2_ at a field of 30 kOe. Measured molar susceptibility as black dots, inverse as blue dots on secondary axis, and Curie–Weiss fit in orange. Neel‐type transition in NdReN_2_ is highlighted with a *T*_N_ = 15.5 K.

The susceptibility of PrReN_2_ was recorded at a field of 30 kOe in the range from 2 to 300 K (Figure 2a). The susceptibility at higher temperatures was modelled with a Curie–Weiss‐fit between 100 and 300 K, yielding an effective magnetic moment of *µ*_eff_ = 3.750(4) *µ*_B_ and a Weiss constant of *θ* = −45.2(4) K. The effective moment is increased with respect to the theoretical *µ*_eff_ = 3.58 *µ*_B_ of Pr^3+^ while a large negative Weiss constant indicates antiferromagnetic exchange interactions. A contribution to the magnetic moment from Re is unlikely owing to the calculated metallic nature of the *Ln*ReN_2_ materials (DFT section). Measurements of the resistivity were, however, not possible as the samples could not be sintered due to decomposition. The deviation of the Curie–Weiss model from the free‐ion value reflects the influence of the surrounding ligand field on the Pr^3+^ ions. The crystal field splits the ^3^H_4_ free‐ion ground state into a non‐magnetic singlet *M*_J_ = 0 ground state followed by a series of doublets and triplets as shown by *Penney* and *Schlapp*.^[^ ^42^ ^]^ The *M*_J_ = 0 ground state leads to a plateau in the susceptibility of powder data at low temperatures, while a long‐range ordering is suppressed as also indicated by powder neutron diffraction data obtained at low temperature (Figure S4). A tentative fit of the susceptibility with the *Penney* and *Schlapp* model gave, however, an insufficient representation of the measured data. Incorporating exchange interactions, which are also indicated by DFT calculations (later chapter), by a mean‐field approach used by *Bleaney* for Pr metal (details in Methods)^[^ ^43^ ^]^ reveals that the susceptibility can be modelled as the combination of crystal field and antiferromagnetic exchange interactions resulting in a total crystal field splitting of *Δ* = 155 cm^−1^, an exchange parameter *λ* = −16.53, and a zero temperature susceptibility of 0.0554 cm^3^ mol^−1^ (Figure S5). The crystal field splitting magnitude is similar to reports on related materials, PrN *Δ* = 168 cm^−1^ and Pr_2_ReN_4_ *Δ* = 186 cm^−1^, while the exchange parameter is of a similar magnitude but reverse sign as that reported for Pr metal (*λ* = 15).^[^ ^3^, ^43^, ^44^ ^]^ This model highlights the competition between crystal field and exchange interactions, which are in line with the antiferromagnetic ordering of the isotypic Nd compound.

#### NdReN_2_

Susceptibility measurements of NdReN_2_ at fields of 30 kOe in the range of 2–300 K show Curie–Weiss‐type behavior at high temperatures and a Néel‐type transition at *T*_N_ = 15.5 K (Figure 2b). A Curie–Weiss fit in the range of 100–300 K yielded an effective magnetic moment of *µ*_eff_ = 3.656(2) µ_B_ (theoretical *µ*_eff_ = 3.62 µ_B_) and a Weiss temperature of *θ* = −45.0(2) K, which are in line with an antiferromagnetic ordering at low T. Fitting the Curie‐tail between 2 and 10 K suggests a 1.5% impurity of a Nd^3+^‐containing byproduct, which is visible by small unaccounted reflections visible in the powder patterns (e.g., Figure 1b inset). The linear response of the magnetization in the field‐dependent measurements carried out at 2 and 300 K reflects the paramagnetic and antiferromagnetic behavior of the sample (Figure S6).

**Figure 3:**
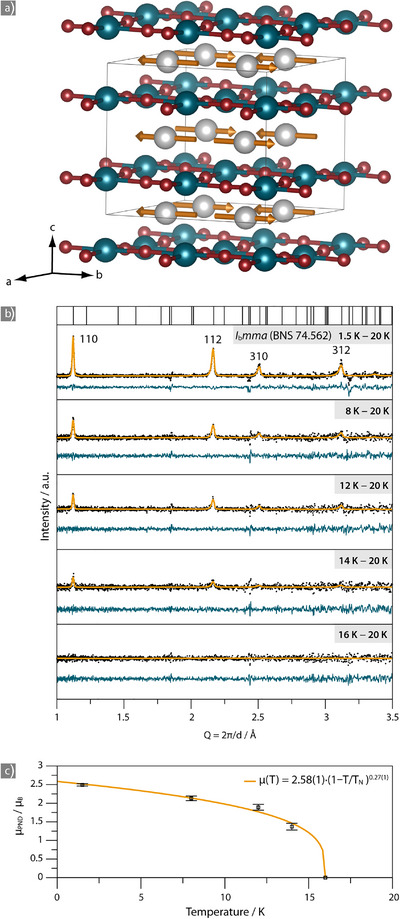
a) Antiferromagnetic structure of NdReN_2_ at a temperature of 1.5 K with Nd in grey, Re in blue, N in red, and magnetic moment vectors in orange. b) Temperature‐dependent refinement results of magnetic reflections based on difference data obtained from subtracting data collected at 20 K. Data are shown as black dots, the fit in orange, the difference curve in blue, the theoretical reflection positions of the magnetic cell indicated as black vertical lines. c) Temperature‐development of the observed magnetic moment with errors as horizontal bars. Mean‐field fit to the data points is displayed in orange and fitting function is given.

The magnetic phase transition was studied by temperature‐dependent collection of powder neutron diffraction data at the WISH beamline of the ISIS Neutron and Muon Source with long scans at 1.5 and 25 K and short scans at 8, 12, 14, 16, and 20 K (absence of magnetic reflections at *T* = 16 K). The magnetic reflections were indexed with a 1 × 1 × 2 supercell of the nuclear structure model (doubling the *c*‐axis). For refinement in the standardized Shubnikov group *I_b_mma* (BNS 74.562, Type IV), the unit cell axes were transformed according to (*a*,*b*,*c*)→(*c*,*b*,*a*). The magnetic structure was initially solved in *P*1 and transformed to Shubnikov group *I_b_mma* with the FINDSYM tool of the Bilbao Crystallographic Server.^[^ ^45^, ^46^ ^]^ This Type IV space group contains a time‐reversal symmetry element along *b*, which leads to antiferromagnetic ordering within neodymium layers that are sandwiched between ReN_4/2_ sheets (Figure 3a), while the magnetic moment vectors are aligned parallel along the stacking direction of [001]. Details of the refinement are provided in Table S7.

Refinement of the magnetic structure against the collected powder neutron data (Figure 3b) gives magnetic moments of 2.49(3), 2.13(6), 1.89(8), and 1.37(3) µ_B_ at 1.5, 8, 12, and 14 K, respectively for the Nd^3+^ sites. The critical behavior of the magnetic phase transition was fitted with a mean‐field‐derived critical expression (Figure 3c), which yielded a zero‐temperature magnetic moment of 2.58(1) µ_B_. The observed magnetic moment is smaller than the expected value of *g J* = 3.27 µ_B_, which reflects the influence of the crystal field, as also reported for similar materials such as ferromagnetic NdN (*µ*_PND_ = 2.69(8) µ_B_) and Nd_2_ReN_4_ (*µ*_PND_ = 2.16(1) µ_B_), and antiferromagnetic NdP (*µ*_PND_ = 1.83(2) µ_B_).^[^ ^3^, ^47^ ^]^ The magnitude of the critical exponent *β* = 0.27(1) is closer to the theoretical Ising model for the 3D (0.312) case than for the 2D (0.125) case.^[^ ^48^, ^49^ ^]^ DFT calculations (details in Supporting Information) reveal direct exchange interactions between Nd–Nd contacts, which, with the likely presence of superexchange, leads to multiple sources of exchange. This is in line with a critical exponent β being in between values of the 3D and 2D Ising models.

### Temperature Dependency of Structure

Both *Ln*ReN_2_ materials were measured with temperature‐dependent powder X‐ray diffraction (Figure S11) at elevated temperatures of 900 °C, while for NdReN_2_ also low‐temperature measurements in the range from 300 to 2 K were performed with powder neutron diffraction (Figure S3). For PrReN_2_ heating and cooling were measured while NdReN_2_ decomposes at 750 °C indicating a lower stability of the Nd‐containing compound, which is in line with the overall decreasing stability of nitride perovskites with heavier lanthanides as suggested by DFT‐based calculations.^[^ ^50^ ^]^ The linear thermal expansion was fitted to the lattice parameters in the range of 70–910 °C for the Pr and from 70 to 690 °C for the Nd compound (for the heating cycle). The results, displayed in Table 1, indicate comparable thermal expansion behavior with other perovskite‐type nitrides such as LaReN_3_ (13 × 10^−6^ K^−1^) and *Ln*_2_ReN_4_ with *Ln* = Pr, Nd (5–10 × 10^−6^ K^−1^).^[^ ^1^, ^3^ ^]^ The coefficient for the *a*‐axis, which is in direction of the short Re–Re contacts, is largest. The displacement of the Re‐positions during heating was refined from the powder patterns for both materials. The distortion of the Re positions from their position in the LaNiO_2_‐type decreases with temperature. However, a phase transition to the aristotype structure is not observed. This indicates a significant strength of the metal−metal interactions.

**Table 1:** Coefficients of linear thermal expansion of *Ln*ReN_2_ determined from temperature‐dependent powder X‐ray diffraction.

Compound	*α*_a_/10^−6^ K^−1^	*α*_b_/10^−6^ K^−1^	*α*_c_/10^−6^ K^−1^	*α*_V_/10^−6^ K^−1^
PrReN_2_	16.5(1)	5.76(7)	5.76(7)	28.2(2)
NdReN_2_	17.1(3)	6.0(1)	6.0(1)	29.3(5)

### Electronic Structure

#### Electronic Structure of NdReN_2_

**Figure 4:**
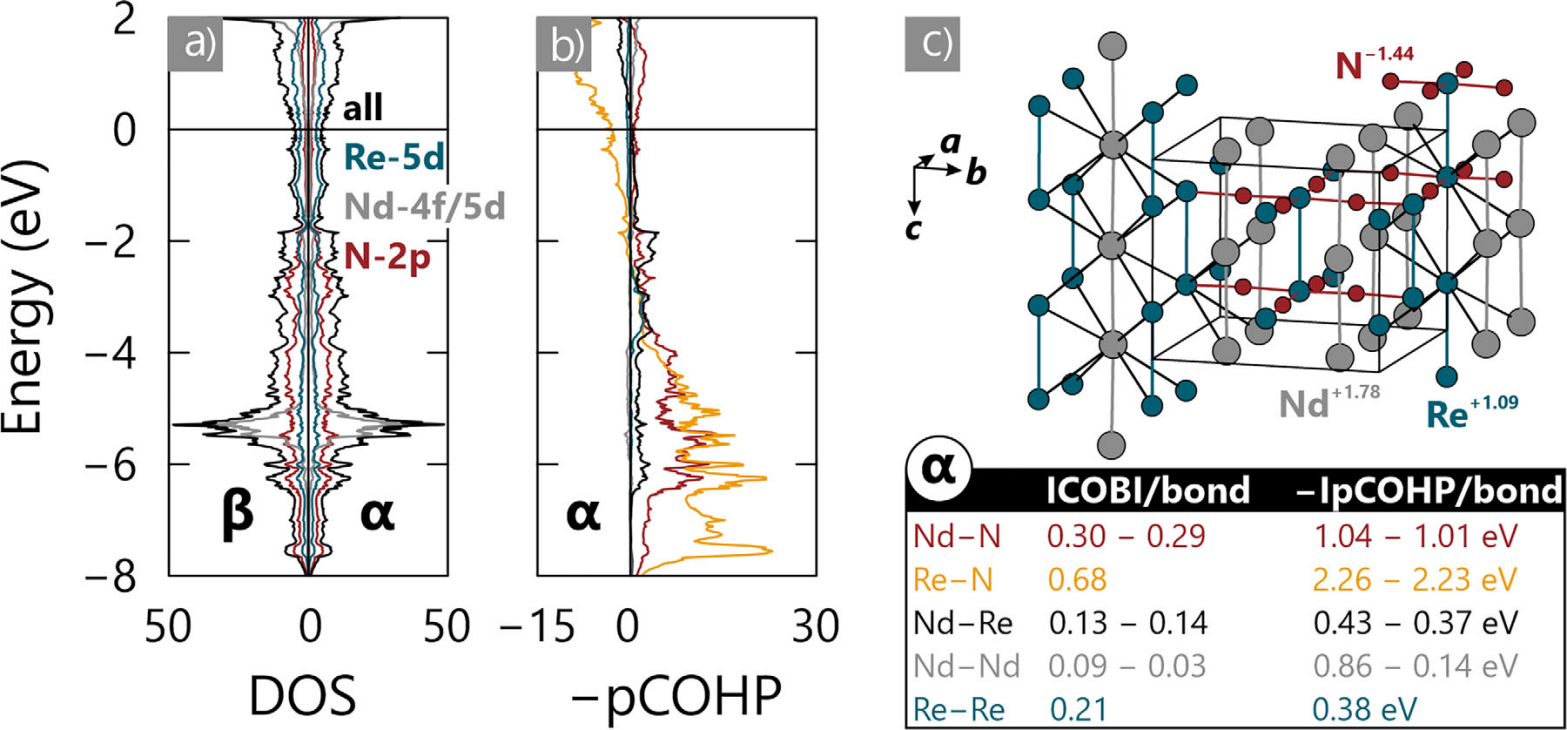
a) Densities‐of‐states (DOS) curve and b) projected crystal orbital Hamilton population (−pCOHP) diagram of NdReN_2_: the Fermi level is represented by the black horizontal line, while the averaged Bader charges and the integrated values of the −pCOHPs as well as crystal orbital bond indices (COBI) have been included in c) showing a representation of the intermetallic network in the nitride. In the case of (c), metal−metal separations being smaller than 3.5 Å have just been included for the benefit of a clear representation, while the lattice setting corresponds to that of the magnetic structure model shown in Figure 3a. In the bonding analysis the results obtained for the α component are provided, which are the same values as those determined for the β component due to the antiferromagnetic ground state, while summation of the results corresponding to both components should approach the outcome of non‐spin‐polarized calculations.

A quantum chemical analysis is carried out to gain insight into the electronic structure of NdReN_2_ (all computational details are provided in Methods in the Supporting Information). The spin‐polarized densities‐of‐states (DOS) curves (Figure 4) reveal that the Fermi level of NdReN_2_ falls in a non‐zero region and is mainly composed of N 2p and Re 5d orbitals and, to a lesser extent, the Nd 5d orbitals, which means that the compound is likely metallic. The calculated Bader (Figure 4c) and Mulliken (Nd: +0.80, Re: +1.07, N: −0.94) charges are in agreement with a metallic state and suggest polar‐covalent M─N bonding as expected from consideration of electronegativity differences. The presence of N 2p states at the Fermi energy suggest traits of a charge‐transfer metal predicted by the Zaanen−Sawatzky−Allen model, which was also found for other transition metal nitrides such as Li_2_Sr[MnN]_2_.^[^ ^51^, ^52^ ^]^

Projected crystal orbital Hamilton populations (pCOHP) and crystal orbital bond indices (COBI) reveal strong Re─N and Nd─N bonding consistent with classic infinite [ReN_4/2_]^3−^ nitridometallate anions (Figure 4c). Significantly, Nd─Nd, Nd─Re, and Re─Re interactions also show substantial ICOBI and IpCOHP values, though smaller than for the M─N bonds due to delocalization. This indicates the simultaneous presence of a cationic intermetallic framework arising from good overlap of the Re and *Ln* valence orbitals.

Additionally, the dimerization of Re atoms exhibits particularly interesting behavior. The Re–Re interactions parallel to the dimerization direction are stronger than the in‐plane interactions (*d*_Re–Re _= 3.95 Å). This creates formal one‐dimensional chains of ReN_4_ moieties along the c‐axis comparable to Krogmann's salt K_2_[Pt(CN)_4_]Cl_0.32_·2.6H_2_O.^[^ ^32^ ^]^ The chains here, however, seem to undergo a partial Peierls‐like distortion through Re_2_ dimer formation as predicted for the famous hydrogen chain by the tight‐binding model or identified at low temperatures in Krogmann's salt K_2_[Pt(CN)_4_]Br_0.3_·3H_2_O.^[^ ^33^, ^34^ ^]^ The Re─Re distances are longer than in the octachloridodirhenate Re_2_Cl_8_^2−^ salts (*d*_Re–Re_ = 2.28 Å) suggesting no quadruple bonding in these materials.^[^ ^31^ ^]^ The equilibrium Re─Re distance likely depends on the dimensionality of the Re─N network (here layers), as shorter Re─Re distances were observed in the chain‐like *Ln*_2_ReN_3_ structures.^[^ ^3^ ^]^

### Comparison of t‐ and o‐LaReN_2_

To investigate the dimerization driving force, a comparison between calculations of the recently reported undistorted tetragonal t‐LaReN_2_ (LaNiO_2_‐type) and a hypothetical orthorhombic o‐LaReN_2_ structure was performed (Figures 1c and S8–S10).^[^ ^1^ ^]^ The La system was chosen as both structure types may exist for the compound (see Supporting Information), while the tetragonal modification was not observed for the Pr and Nd compounds. The orthorhombic distortion, which leads to simultaneous bond stretching (weakening) and bond shortening (strengthening), provides a modest energy gain as indicated by the total ─IpCOHP/f.u. for t‐LaReN_2_ = 34.15 eV and for o‐LaReN_2_ = 34.23 eV.^[^ ^53^ ^]^ In the orthorhombic structure, the Re─Re interactions originate from all d‐orbitals, including minor contributions from the *d*_x2‐y2_ orbitals that are largely involved in Re─N bonding, similar to the bonding in Krogmann's salts. The bonding within the ReN_4_ units (Supporting Information for further discussion) is similar to a square planar complex, while the distortion of the chains yields a slight energy gain of Re─N bonding as indicated by the respective ─IpCOHP values (─IpCOHP/f.u._Re–N_ = 14.83 eV for t‐LaReN_2_ versus 14.94 eV for o‐LaReN_2_). Moreover, an analysis based on the fragmented molecular orbitals of a Re_4_ chain shows that the states originating from d_z2_ orbitals construct σ‐type contributions along the *c*‐axis and, furthermore, split into non‐degenerate states as the Re_4_ chain distorts, indicative of a Jahn–Teller distortion. In the extended solid, the distortion leads to an energy gain of the states involved in Re─Re bonding (─IpCOHP/f.u._Re–Re_ = 0.23 eV for t‐LaReN_2_ versus 0.34 eV for o‐LaReN_2_), and notably a splitting of the Re‐5d_z2_ band into bonding and antibonding branches and a depletion of states in between (Figure S10). This has also been observed in the embedded Peierls distortion in MoO_2_, where metal–metal pairing, via the face of anion polyhedra, create chains of long and short Mo–Mo interactions.^[^ ^54^, ^55^ ^]^ A band gap does not open up in o‐ and t‐LnReN_2_ as indicated by populated states at the Fermi level corresponding to the (intermetallic) framework.^[^ ^56^, ^57^, ^58^, ^59^ ^]^ Moreover, the temperature‐dependent powder X‐ray diffraction suggests that the Re─Re bonding is stable up to high temperatures (Figure S11). The here‐observed dimerization is therefore similar to rutile‐related oxides such as VO_2_ and especially MoO_2_, which also shows no phase transition and remains metallic over a large temperature range.^[^ ^60^ ^]^

## Conclusion

The herein shown stabilization of LaNiO_2_‐type nitrides increases the compositional and structural space of the recently established class of nitride perovskites and related materials. Both o‐*Ln*ReN_2_ (*Ln* = Pr, Nd) materials were prepared with ammonium azide to create reducing synthesis conditions under high pressures that opened the pathway to anion‐vacancy ordered perovskite‐related nitrides of the LaNiO_2_‐type. While the Pr compound shows no magnetic ordering, the Nd compound is an antiferromagnet. DFT calculations gave further insight into additional direct Nd–Nd exchange, which probably contributes to the antiferromagnetic state close to a 3D Ising model. The most prominent structural feature in the o‐*Ln*ReN_2_ compounds is the Peierls‐like distortion of Re‐chains leading to Re_2_ dimerization, which is similar to structural features observed in simple rutile‐type transition metal oxides such as MoO_2_. Such a dimerization was not observed for LaNiO_2_, hence the *Ln*ReN_2_ series may show a tunable Peierls‐type distortion dependent on *Ln* cation size that will be explored in future research. Moreover, the calculations indicated that both the undistorted t‐LaNiO_2_ aristotype LaReN_2_ as well as the o‐*Ln*ReN_2_ compounds comprise not only extended nitridometallate anions but also an extended cationic *Ln* network interconnected with linear [ReN_4_]_∞_ chains. This makes the structural chemistry of the *Ln*ReN_2_ materials a border case between nitridometallates, interstitial nitrides, cluster and complex chemistry. Especially, the connection to Krogmann's salts is highlighted by the 1D nature of the Re–Re interactions. As the Re–Re interatomic distance is likely controlled by the 2D layered structures, the preparation of further materials with different dimensionalities and smaller transition metal centers such as W, Mo, and Nb is intriguing and may lead to a full Peierl's distortion with electron localization in analogy to octachloridodirhenate(III) Re_2_Cl_8_^2−^ anions.

## Supporting Information

The authors have cited additional references within the Supporting Information.^[^ ^61^, ^62^, ^63^, ^64^, ^65^, ^66^, ^67^, ^68^, ^69^, ^70^, ^71^, ^72^, ^73^, ^74^, ^75^, ^76^, ^77^, ^78^, ^79^, ^80^, ^81^, ^82^, ^83^, ^84^, ^85^, ^86^, ^87^, ^88^, ^89^, ^90^, ^91^ ^]^

## Conflict of Interests

The authors declare no conflict of interest.

## Supporting information



Supporting Information

Supporting Information

## Data Availability

Data for this article, including diffraction data and magnetization data, are available at Open Access LMU under DOI: https://doi.org/10.5282/ubm/data.753. The magnetic crystallographic information file (mcif) of NdReN_2_ will be made available on the Bilbao Crystallographic Server (MAGNDATA).
