# Supplementary material for: Possible Peierls Distortion Through Re2 Dimer Formation in the LaNiO2‐Type Nitrides LnReN2 (Ln = Pr, Nd)
Source: Angew Chem Int Ed Engl. 2025 Dec 31;65(5):e19710. doi: 10.1002/anie.202519710 (PMC12851013; doi:10.1002/anie.202519710)
Supplement: Supplementary file 1 — Supporting Information [file ANIE-65-e19710-s002.pdf]

# Table of Contents


Experimental Methods and Analysis ........ 2

Preparation ........ 2

Diffraction ........ 2

Physical Properties Measurements ........ 3

Electron microscopy and energy dispersive X-ray spectroscopy ........ 3

Computational Details ........ 3

Scanning Electron Microscopy ........ 5

Powder Neutron Diffraction ........ 6

Magnetic Powder Neutron Diffraction ........ 12

Magnetic Properties ........ 14

Structural refinement of $LaReN_2$ ........ 15

Additional information on DFT-calculations of $NdReN_2$ and t-/o-$LaReN_2$ ........ 16

References ........ 21

# Experimental Methods and Analysis

## Preparation

**$LnReN_2$.** Samples of $LnReN_2$ were prepared through high-pressure high-temperature reaction at 8 GPa and ca. 1100 °C. Reaction was carried out between rare earth nitrides (5 wt-% surplus), Re powder (Alfa Aesar, 99.99 %) and ammonium azide $NH_4N_3$ according to following Eq. 1:

$$LnN + Re + NH_4N_3 \rightarrow LnReN_2 + 2\ H_2 + 3/2\ N_2$$

Samples were obtained as black microcrystalline powders with ca. 50 mg yield per experiment. Small amounts of Re by-product was detected in powder X-ray diffraction in some samples.

The high-pressure conditions were achieved with a multianvil large-volume 1000t hydraulic press build by Voggenreiter (Mainleus, Germany). The press was operated with an octahedron-within-cubes payload residing inside a Walker-type module. Co-doped tungsten carbide cubes with 11 mm edge-truncation served as second stage anvils and 18 mm edge $Cr_2O_3$-doped MgO-octahedra as pressure transmitting medium. The starting materials were ground in a glovebox (< 1 ppm $O_2$/$H_2O$, MBraun, Garching, Germany) and then tightly packed in a Zr-foil inlay (0.025 mm thickness, 99.9%, Thermo Scientific Chemicals ), which was then placed inside an h-BN crucible, that was previously treated by boiling in methanol to remove boric acid. The samples were prepared by a 60/120/30 heating profile (ramp up, dwell, ramp down).

**LnN.** The rare earth mononitrides were prepared by heating freshly filed Pr and Nd metal shavings (99.9 %, Alfa Aesar) under dry dinitrogen atmosphere at ca. 1200 °C for 18 h. The reaction conditions were achieved in a radiofrequency furnace and samples were residing in clean tungsten crucibles.

**$NH_4N_3$**. Ammonium azide was prepared from reaction of $NaN_3$ (99.99 %, Sigma Aldrich) and $NH_4NO_3$ (99.99 % Sigma Aldrich) at 180 °C in an open container in air.

**Disclaimer.** Azides are highly toxic and can cause cancer while being soluble in water and stable in air. Azides can combust at elevated temperatures. Handle with care. Sodium azide decomposes to form sodium and dinitrogen.

## Diffraction

**Powder X-ray diffraction.** Powder X-ray diffraction data were obtained on a Stoe StadiP powder diffractometer (Stoe & Cie, Darmstadt, Germany) equipped with an Ag X-ray source, a Germanium(111) single crystal to filter $K_{\alpha 1}$ radiation, and a MYTHEN 1K Si-strip detector (Dectric, Baden, Switzerland). Samples were diluted with amorphous boron and loaded into glass capillaries (0.5 mm outer diameter, Hilgenberg, Malsfeld, Germany). The Rietveld refinements were done with TOPAS Academic V6.[1]

Temperature-dependent powder X-ray diffraction data were collected with a Stoe StadiP powder diffractometer equipped with a graphite furnace and an image-plate position sensitive detector. Data were collected starting at 60 °C in steps of 20 °C up to a maximum temperature of 900 °C. For $PrReN_2$, a cooling run was performed with equal steps. Samples were loaded into quartz capillaries (0.4 mm outer diameter, Hilgenberg, Malsfeld, Germany) in a glovebox and measured under exclusion of atmosphere.

**Powder Neutron diffraction.** Neutron powder diffraction data of $PrReN_2$ was collected on the D20 beamline of the Institute Laue Langevin (Grenoble, France). High-resolution data using a take-off angle of 90° and a wavelength of 1.54 Å were collected at 300 K, while high-flux measurements with a take-

off angle of 42° and a wavelength of 2.4 Å were collected at 2 and 50 K. The latter measurements were done to check for the presence of magnetic ordering in the material, which was however not the case.

Neutron powder diffraction data of $NdReN_2$ was collected on the WISH beamline of the ISIS Muon and Neutron Source (Oxfort, UK). Long scans at 300, 25, and 1.5 K were collected and a series of short scans at 8, 12, 14, 16, 20, 25, 50, 100, 200 K. Refinements were performed as follows: Co-refinement of long-scan 300 K data of detector bank 5 with PXRD data, refinement of long-scans at 25 and 1.5 K data on Banks 2-5 (PND only). Short scans were used to extract lattice parameters without structural information and magnetic moments in case $T < T_N$.

Crystal structures were visualized using VESTA.[2]

## Physical Properties Measurements

Magnetic properties of $LnReN_2$ were measured with a vibration sample magnetometer option for a Quantum Design Physical Property Measurement System (PPMS, Sand Diego, CA, USA). Susceptibility data were recorded in a field of 30 kOe in a temperature range between 2 and 300 K. Field-dependent measurements were conducted at 2 and 300 K in fields between ±50 kOe. Samples were placed inside polyethylene capsules of known diamagnetic contribution. Susceptibility measurements run in 100 Oe fields yielded qualitatively similar results and did not show any other magnetic features.

The Penney-Schlapp analysis is based on the splitting of the fit $^3H_4$ multiplet of $Pr^{3+}$:$4f^2$ in a cubic crystal field. Penney and Schlapp calculated the energy levels of each state as a function of a parameter *a* and the magnetic field *H*. From these they obtain with Boltzmann statistics an expression for the susceptibility that is only dependent on *a*. The susceptibility for a cubic crystal field is:

$$X_{PS} = \frac{2N_A g^2 \mu_B^2}{a} \cdot \frac{\left[\frac{53e^{13u}}{5760} + \frac{e^{-2u}}{30} - \frac{61e^{-7u}}{2688} - \frac{5e^{-14u}}{252} + \frac{u(25e^{13u}+e^{-7u})}{192}\right]}{3e^{13u}+2e^{-2u}+3e^{-7u}+e^{-14u}} \quad \text{where } u = \frac{48a}{kT} \quad .$$

Here, $N_A$ is Avogadro's number, *g* is the Lande-factor (= 4/5 for $Pr^{3+}$), and $\mu_B$ the Bohr magneton. Bleaney calculated the contribution of exchange interactions as $\frac{1}{X} = \frac{1}{X`} - \lambda$, where X is the actual susceptibility, and X' is the susceptibility without exchange λ. Negative values of λ indicate antiferromagnetic exchange interactions.

## Electron microscopy and energy dispersive X-ray spectroscopy

Morphology and chemical composition were investigated with a Helios Nanolab G3 DualBeam (UC configuration; FEI) equipped with a X-Max 80 silicon drift detector (Oxford Instruments). Samples were sputtered with carbon to decrease electrical charging.

## Computational Details

In order to provide an insight into the electronic structure scenario, analysis backed by density functional theory-based means have been accomplished. In this context, the electronic structure has been analysed for $NdReN_2$, as the outcome of the neutron diffraction experiments provided adequate information that could be used to construct a magnetic structure model as starting parameter for the spin-polarized elec-

tronic structure calculations. Furthermore, the electronic structures were analysed for the recently reported t-$LaReN_2$ and a hypothetical o-$LaReN_2$, whose structure model has been developed based on that of $NdReN_2$, in order to evaluate the structural preferences of the nitrides.[3]

All electronic structure computations including full structural optimizations of the lattice parameters and atomic positions were accomplished using the projector augmented wave (PAW) method[4] as implemented in the Vienna ab initio simulation package[5-9] (VASP). Correlation and exchange were depicted by means of the generalized gradient approximation[10] (GGA−PBE), while an effective on-site Coulomb interaction term that was added to the Kohn-Sham Hamiltonian in order to describe the strong correlations within the Nd-4f states in the case of the computations of the neodymium-containing species. A preliminary screening considering a range of different $U_{\mathrm{eff}}$ parameter indicated that a value of 7.00 eV best describes the positions and features expected[11] for the Nd-4f states. The energy cut-off of the plane wave basis set was 500 eV, while sets of 7 × 7 × 8 and 14 × 14 × 16 **k**-points were used to sample the first Brillouin zones of o-$LnReN_2$ (Ln: La, Nd) and t-$LaReN_2$, respectively. Finally, the computations were expected to be converged, as the energy difference between two iterative steps fell below a value of up to $10^{-8}$ eV (and $10^{-6}$ eV) for the electronic (and ionic) relaxation.

In framework of the electronic structure analysis, we also investigated the nature of bonding for the ternary nitrides. In doing so, we determined the projected crystal orbital Hamilton populations[12-14] (pCOHP), the crystal orbital bond indices[15] (COBI), their respective integrated values and the Mulliken[16] as well as Bader[17] charges. The Bader charges were obtained from the results of the plane wave-based computations with the aid of the program[18-21] bader, while the construction of the other bonding indicators required the use of local basis sets, whose nature is in stark contrast to that of the plane waves utilized in the computations. Therefore, the results of the plane wave-based calculations had to be transferred to the all Slater-type orbital with the aid of transfer matrices. This transfer was achieved by using the Local Orbital Basis Suite Towards Electronic-Structure Reconstruction[22-24] (LOBSTER) program, while the wxDragon code[25] was used for further analyses and visualization of the results. Finally, the representation showing the intermetallic network within the crystal structure of $NdReN_2$ was created using the program[26] DIAMOND. In order to provide an insight into the origin of the structural distortions observed for the orthorhombic structure models, we also determined the Fragment Molecular Orbitals and Molecular Orbital Formation Energy (MOFE),[27] i.e. the MO-based analogue of the pCOHP, for $ReN_4$ and $Re_4$ units in o- as well as t-$LaReN_2$. The FMOs were constructed from the results of the plane wave-based computations with the aid of the LOBSTER code, while the FMOs were visualized by using the program VESTA.

## Scanning Electron Microscopy

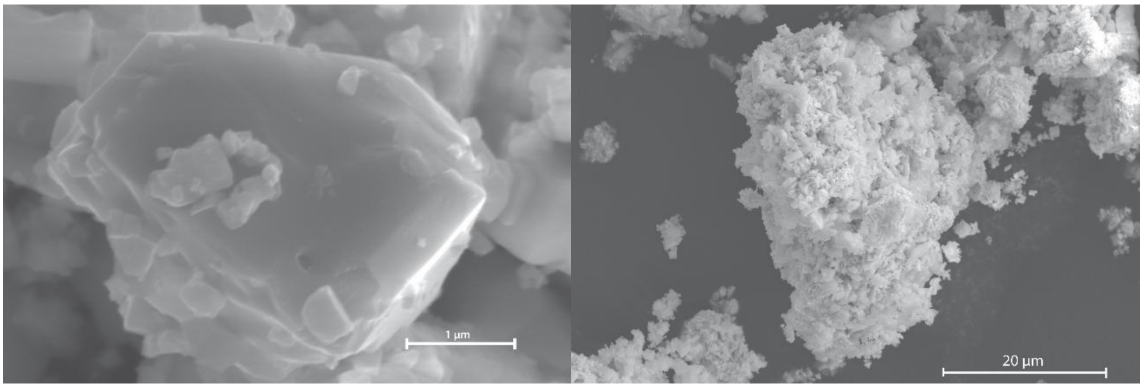


**Figure S1:** Scanning electron microscopy image of $PrReN_2$ (left) and $NdReN_2$ (right).

**Table S1:** EDX data collected on $PrReN_2$ and $NdReN_2$. Oxygen content (on the 5 % level for Pr, 20 % level for Nd) was excluded.

| Measurement | N / at-% | Ln / at-% | Re / at-% |
|---|---|---|---|
| **$PrReN_2$** | | | |
| **1** | 47.2 | 26.3 | 26.6 |
| **2** | 44.0 | 27.3 | 28.6 |
| **3** | 49.4 | 25.0 | 25.6 |
| **4** | 57.3 | 21.4 | 21.3 |
| **5** | 54.2 | 22.6 | 23.2 |
| **6** | 53.4 | 23.2 | 23.5 |
| **7** | 47.0 | 26.4 | 26.6 |
| **8** | 53.9 | 23.0 | 23.0 |
| **9** | 52.8 | 23.2 | 23.9 |
| **Mean, normalized on Re** | 2.07 | 0.98 | 1.00 |
| **Standard Deviation** | 0.17 | 0.08 | 0.09 |
| | | | |
| **$NdReN_2$** | | | |
| **1** | 49.4 | 27.0 | 23.6 |
| **2** | 52.7 | 25.4 | 21.9 |
| **3** | 51.8 | 24.7 | 23.6 |
| **4** | 55.3 | 23.3 | 21.4 |
| **5** | 63.5 | 19.6 | 16.9 |
| **6** | 58.3 | 21.1 | 20.7 |
| **Mean, normalized on Re** | 2.58 | 1.10 | 1.00 |
| **Standard Deviation** | 0.20 | 0.11 | 0.10 |
| | | | |

## Powder Neutron Diffraction

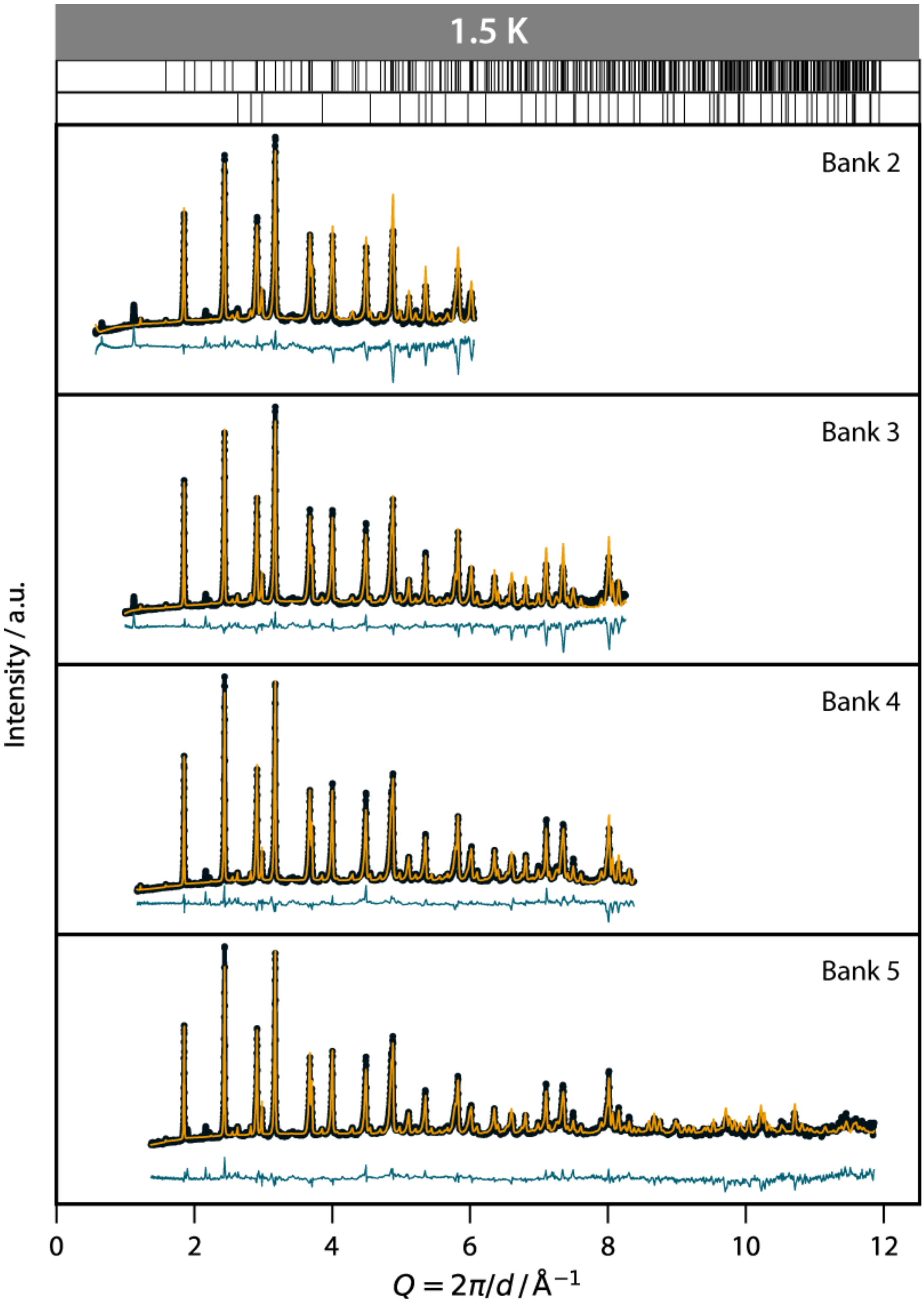

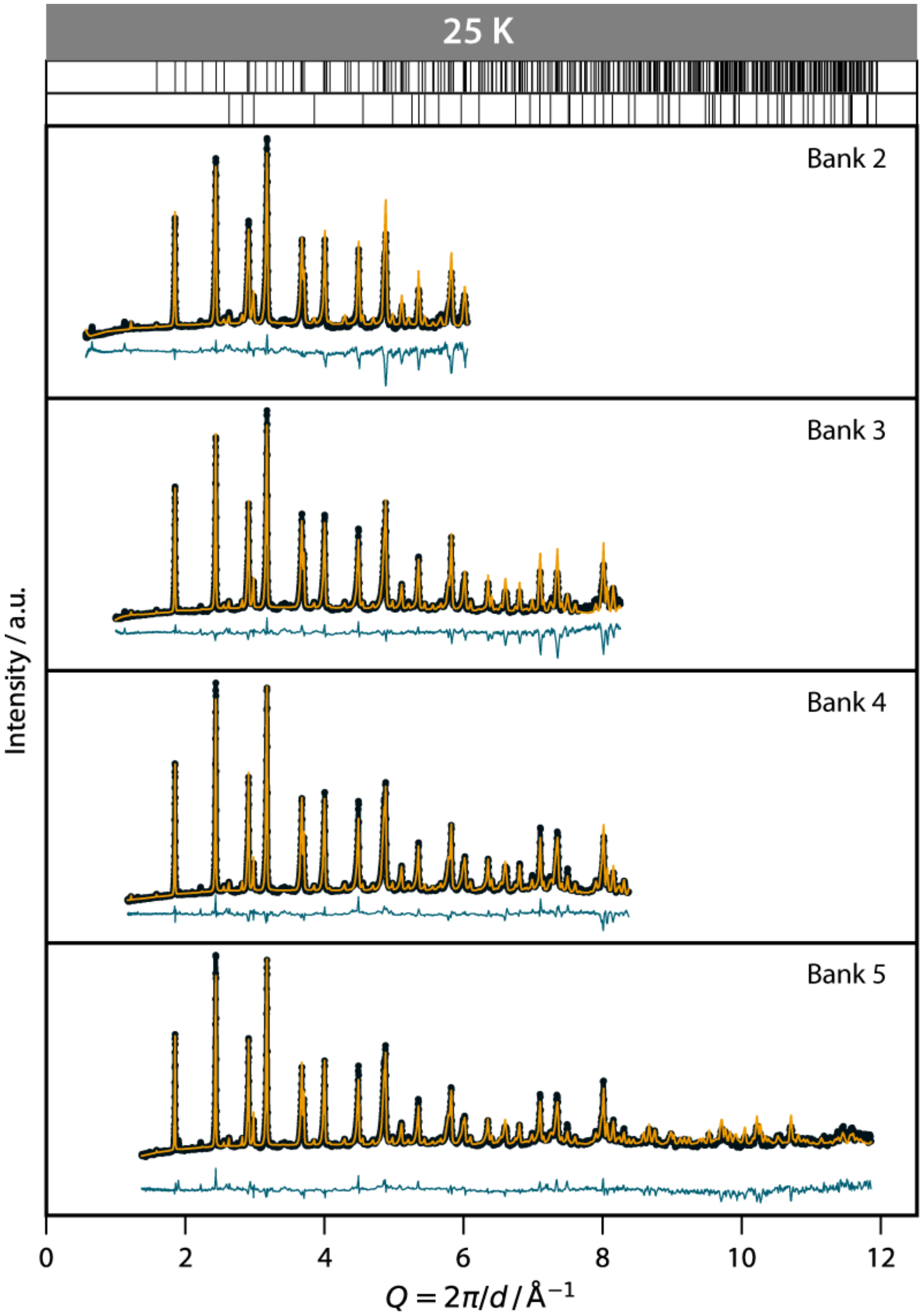


**Figure S2:** Rietveld refinements of powder neutron diffraction data at 1.5 and 25 K collected on $NdReN_2$. Refinement in orange, data as black circles, difference as blue line. Bragg positions are indicated as vertical lines (top one from $NdReN_2$, bottom one from a ~4 w-% Re impurity).

**Table S2:** Crystallographic information for $PrReN_2$ from corefinemnt of PND and PXRD data at 300 K.

| | | |
|---|---|---|
| **Crystal data** | | |
| Formula | $PrReN_2$ | |
| Formula mass, g·mol$^{-1}$ | 355.14 | |
| Crystal system | orthorhombic | |
| Space group | *Cmmm* (no. 65) | |
| Cell parameters, Å | $a$ = 6.85813(11) | |
| | $b$ = 7.9293(6) | |
| | $c$ = 3.9652(3) | |
| Cell volume, Å$^3$ | 215.63(2) | |
| Z | 4 | |
| F(000) | 592 | |
| Calc. density, g·cm$^{-3}$ | 10.940 | |
| **Data collection** | | |
| Diffractometer | ILL D20 High Intensity Beamline | Stoe & Cie StadiP |
| Radiation | Neutron, CW (λ = 1.54 Å) | Ag-$K_{\alpha 1}$ |
| Temperature, K | 300 | |
| Q-range | 0.71, 7.77 | 0.58, 13.73 |
| Number of Reflections | 141 | 715 |
| **Overall Refinement** | | |
| GOF | 0.033 | |
| $R_p$, $R_{wp}$, $R_{exp}$ | 0.058,<br>0.040,<br>0.012 | |
| $R_{Bragg}$ | 0.019 | |

**Table S3:** Crystallographic information for $NdReN_2$ from corefinemnt of PND and PXRD data at 300 K.

| | | |
|---|---|---|
| **Crystal data** | | |
| Formula | $NdReN_2$ | |
| Formula mass, g·mol$^{-1}$ | 358.47 | |
| Crystal system | orthorhombic | |
| Space group | *Cmmm* (no. 65) | |
| Cell parameters, Å | $a$ = 6.80455(12) | |
| | $b$ = 7.9179(4) | |
| | $c$ = 3.9595(2) | |
| Cell volume, Å$^3$ | 213.33(2) | |
| Z | 4 | |
| F(000) | 596 | |
| Calc. density, g·cm$^{-3}$ | 11.161 | |
| **Data collection** | | |
| Diffractometer | ISIS WISH Long-Wavelength Diffractometer | Stoe & Cie StadiP |
| Radiation | Neutron, Time-of-flight | Ag-$K_{\alpha 1}$ |
| Temperature, K | 300 | |
| Bank | Detector Bank 5 | |
| Q-range | 1.37, 11.86 | 0.39, 13.73 |
| Number of Reflections | 466 | 710 |
| **Overall Refinement** | | |
| GOF | 0.028 | |
| $R_p$, $R_{wp}$, $R_{exp}$ | 0.064,<br>0.054,<br>0.019 | |
| $R_{Bragg}$ | 0.029 | |

**Table S4:** Crystallographic information for $NdReN_2$ from PND data at 25 and 1.5 K.

| | | |
|---|---|---|
| | **Crystal data** | |
| Formula | $NdReN_2$ | |
| Formula mass, g·mol$^{-1}$ | 358.47 | |
| Crystal system | orthorhombic | |
| Space group | *Cmmm* (no. 65) | |
| Cell parameters, Å | *a* = 6.77711(9)<br>*b* = 7.9095(10)<br>*c* = 3.9551(5) | *a* = 6.77652(11)<br>*b* = 7.9086(9)<br>*c* = 3.9554(4) |
| Cell volume, Å$^3$ | 212.01(4) | 211.98(3) |
| Z | 4 | |
| F(000) | 596 | |
| Calc. density, g·cm$^{-3}$ | 11.231 | 11.232 |
| | **Data collection** | |
| Diffractometer | ISIS WISH Long-Wavelength Diffractometer | |
| Radiation | Neutron, Time-of-flight | |
| Temperature, K | 25 | 1.5 |
| Number of Banks | 4 | |
| | **Refinement** | |
| GOF | 0.046 | 0.046 |
| $R_p$, $R_{wp}$, $R_{exp}$ | 0.042,<br>0.046,<br>0.010 | 0.046,<br>0.054<br>0.010 |
| $R_{Bragg}$ | 0.038 | 0.038 |

**Table S5:** Atomic positions, occupation, isotropic displacement parameters of $LnReN_2$ from PND/PXRD data at given temperatures.

| Temperature / K | Atom | *x* | *y* | *z* | Occ | $B_{iso}$ / Å$^2$ | Site | Sym. |
|---|---|---|---|---|---|---|---|---|
| **$PrReN_2$** | | | | | | | | |
| **Co-Ref. 300** | Re1 | 0.22761(14) | 0 | 0.5 | 1 | 0.0026(2) | 4h | 2mm |
| | Pr1 | 0 | 0.2611(3) | 0 | 1 | 0.0037(4) | 4i | m2m |
| | N1 | 0.25 | 0.25 | 0.5 | 1 | 0.0071(3) | 4f | ..2/m |
| | N2 | 0.2344(3) | 0 | 0 | 1 | 0.0071(3) | 4g | 2mm |
| **$NdReN_2$** | | | | | | | | |
| **Co-Ref. 300** | Re1 | 0.22941(13) | 0 | 0.5 | 1 | 0.0025(2) | 4h | 2mm |
| | Nd1 | 0 | 0.2603(2) | 0 | 1 | 0.0024(3) | 4i | m2m |
| | N1 | 0.25 | 0.25 | 0.5 | 1 | 0.0089(5) | 4f | ..2/m |
| | N2 | 0.2366(7) | 0 | 0 | 1 | 0.0089(5) | 4g | 2mm |
| **25** | Re1 | 0.2338(4) | 0 | 0.5 | 1 | 0.0025(4) | 4h | 2mm |
| | Nd1 | 0 | 0.2612(4) | 0 | 1 | 0.0028(5) | 4i | m2m |
| | N1 | 0.25 | 0.25 | 0.5 | 1 | 0.0080(4) | 4f | ..2/m |
| | N2 | 0.2282(7) | 0 | 0 | 1 | 0.0080(4) | 4g | 2mm |
| **1.5** | Re1 | 0.2340(5) | 0 | 0.5 | 1 | 0.0024(5) | 4h | 2mm |
| | Nd1 | 0 | 0.2607(5) | 0 | 1 | 0.0023(6) | 4i | m2m |
| | N1 | 0.25 | 0.25 | 0.5 | 1 | 0.0080(5) | 4f | ..2/m |
| | N2 | 0.2285(8) | 0 | 0 | 1 | 0.0080(5) | 4g | 2mm |

**Table S6:** Interatomic distances (in Å) occurring in $LnReN_2$ from PND/PXRD data at given temperatures.

| **Atom Pair** | **$PrReN_2$** | **$NdReN_2$** | | |
|---|---|---|---|---|
| | | | | |
| Temperature / K | **Co-Ref. 300** | **Co-Ref. 300** | 25 | 1.5 |
| Re1–N1 | 1.9832(2) | 1.9804(2) | 1.9804(3) | 1.9801(3) |
| Re1–N2 | 1.9832(2) | 1.9804(2) | 1.9779(3) | 1.9781(3) |
| Re1–N2 | 1.9883(2) | 1.9844(1) | 1.9779(3) | 1.9781(3) |
| Re1–N1 | 1.9883(2) | 1.9844(1) | 1.9804(3) | 1.9801(3) |
| | | | | |
| Ln1–N1 | 2.621(3) | 2.610(4) | 2.6056(3) | 2.6055(3) |
| Ln1–N2 | 2.621(3) | 2.610(4) | 2.581(4) | 2.579(5) |
| Ln1–N1 | 2.6226(2) | 2.6115(1) | 2.6056(3) | 2.6055(3) |
| Ln1–N2 | 2.6226(2) | 2.6115(1) | 2.581(4) | 2.579(5) |
| Ln1–N1 | 2.6226(2) | 2.6115(1) | 2.6056(3) | 2.6055(3) |
| Ln1–N1 | 2.6226(2) | 2.6115(1) | 2.6056(3) | 2.6055(3) |
| Ln1–N2 | 2.628(3) | 2.615(4) | 2.638(4) | 2.639(5) |
| Ln1–N2 | 2.628(3) | 2.615(4) | 2.638(4) | 2.639(5) |
| | | | | |
| Ln1–Ln1 | 3.4336(3) | 3.4062(2) | 3.3932(5) | 3.3925(5) |
| Re1–Re1 | 3.122(2) | 3.122(2)d | 3.169(6) | 3.171(7) |

## Addition to the structure discussions

The rare earth atoms are coordinated in a distorted $LnN_8$ cube with $d_{Pr–N}$ = 2.610(7) to 2.644(7) Å and $d_{Nd–N}$ = 2.569(5) to 2.655(5) Å. These distances are in agreement with other rare earth-containing nitrides with cubic $LnN_8$ polyhedra such as in $Ln_3B_2N_4$ ($d_{Pr–N}$ = 2.66 Å, $d_{Nd–N}$ = 2.65 Å) or square antiprismatic $LnN_8$ polyhedra such as in $Ln_3Si_6N_{11}$ ($d_{Pr–N}$ = 2.60 Å, $d_{Nd–N}$ = 2.62 Å).[28-29]

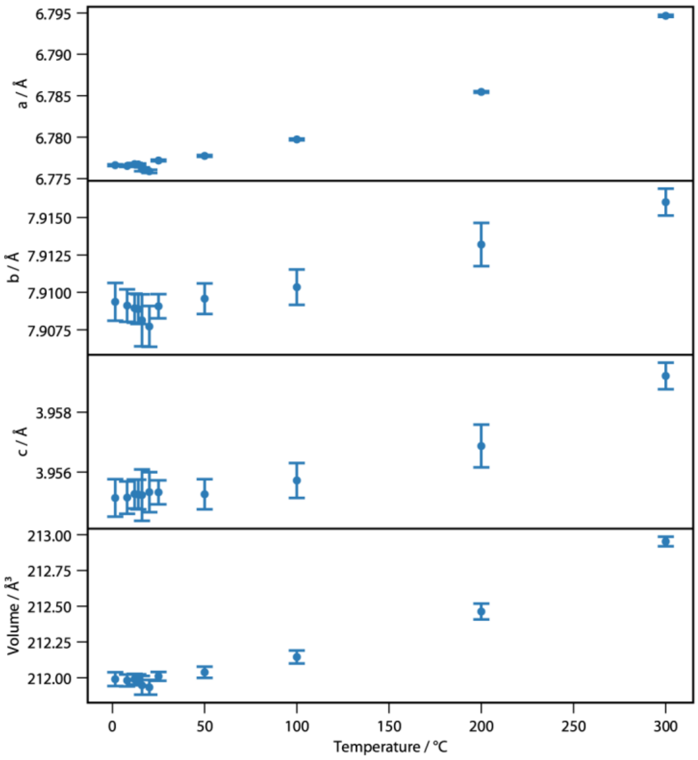


**Figure S3:** Development of lattice parameters of $NdReN_2$ while cooling from room temperature to 1.5 K in following steps: 8, 12, 14, 16, 20, 25, 50, 100, 200 K. Data obtained from PND data collected at the WISH-beamline. The data show a decrease in lattice parameters for all three axes.

# Magnetic Powder Neutron Diffraction

**Table S7:** Crystallographic information for magnetic structure refinement of $NdReN_2$ from PND data at 1.5K.

| | | | | |
|---|---|---|---|---|
| Compound | **$NdReN_2$** | | | |
| Parent space group | *Cmmm* (no. 65) | | | |
| Propagation vector(s) | (0, 0, ½) | | | |
| Transformation from parent basis to the one used | (**2c**, **b**, **a**; 0, 0,0) | | | |
| MSG symbol | $I_bmma$ | | | |
| MSG number | 74.562 | | | |
| Transformation from basis used to standard setting of MSG | (**a**, **b**, **c**; 0, 0,0) | | | |
| Magnetic point group | mmm1' | | | |
| Unit-cell parameters (Å, °) | *a* = 7.906<br>*b* = 7.907<br>*c* = 6.7743, | | *α* = 90<br>*β* = 90<br>*γ* = 90 | |
| MSG symmetry operations | x, y, z, +1<br>x, −y, −z, +1<br>−x, y+1/2, −z, +1<br>−x, −y+1/2, z, +1<br>−x, −y, −z, +1<br>−x, y, z, +1<br>x, −y+1/2, z, +1<br>x, y+1/2, −z, +1 | | {1 \| 0, 0, 0}{$2_{[100]}$ \| 0, 0, 0}<br>{$2_{[010]}$ \| 0, 1/2, 0}<br>{$2_{[001]}$ \| 0, 1/2, 0}<br>{−1 \| 0, 0, 0}<br>{$-2_{[100]}$ \| 0, 0, 0}<br>{$-2_{[010]}$ \| 0, 1/2, 0}<br>{$-2_{[001]}$ \| 0, 1/2, 0} | |
| MSG symmetry centering operations | x, y, z, +1<br>x+1/2, y+1/2, z+1/2, +1<br>x, y+1/2, z, −1<br>x+1/2, y, z+1/2, −1 | | {1 \| 0, 0, 0}<br>{1 \| 1/2, 1/2, 1/2}<br>{1' \| 0, 1/2, 0}<br>{1' \| 1/2, 0, 1/2} | |
| Positions of magnetic atoms | Nd1 | 0.25970 | 3/4 | 0 |
| Positions of non-magnetic atoms | N1<br>N2<br>Re1 | 1/4<br>0<br>0 | 0<br>3/4<br>0 | 1/4<br>0.77120<br>0.76540 |
| Magnetic moment components (μB) of magnetic atoms, symmetry constraints and moment magnitudes | Nd1 | (0, 2.490, 0) | (0, $m_y$, 0) | 2.490 |

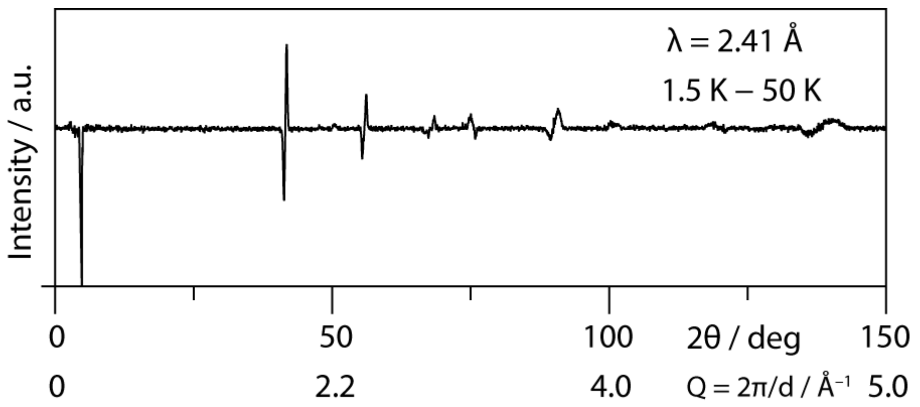


**Figure S4:** Difference powder neutron diffraction data collected on $PrReN_2$ at temperatures of 1.5 and 50 K. Data were obtained at the D20 beamline of the ILL in high-flux mode with a wavelength of 2.41 Å. The mismatch in intensities reflect structural changes owed to changes in lattice parameters. The spike at 5° stems from a faulty detector cell.

## Magnetic Properties

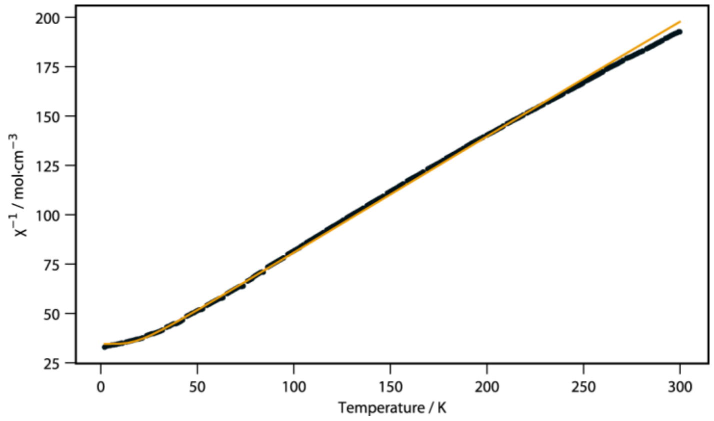


**Figure S5:** Penney-Schlapp fit with antiferromagnetic exchange interaction to the inverse of the susceptibility of $PrReN_2$ collected at 30 kOe. A constant contribution to the magnetisation $X_0 = 2.10 \pm 0.18 \cdot 10^{-4}$ $cm^3$/mol was included. The results of the fit gave a crystal field parameter $a = -0.120 \pm 0.001$ $cm^{-1}$ and $\lambda = -16.53$.

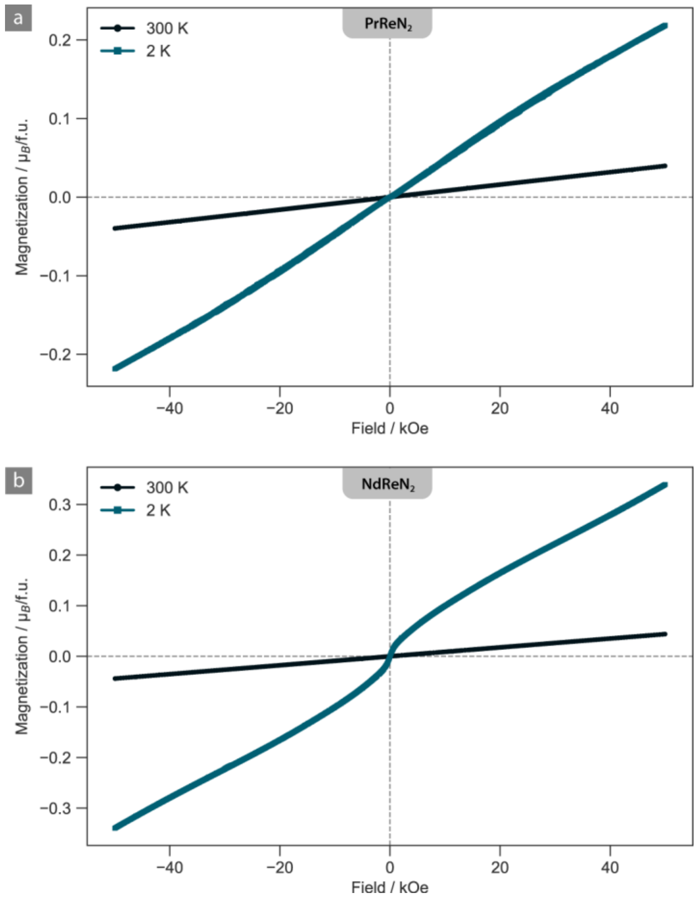


**Figure S6:** Magnetisation measurements carried out on **(a)** $PrReN_2$ and **(b)** $NdReN_2$ between −50 to 50 kOe, 300 K in black, 2 K in blue.

## Structural refinement of $LaReN_2$

The powder X-ray data obtained on $LaReN_2$ (published by Kloß et al.)[3] were re-examined for a possible Re–Re interaction. The poor data-quality resulting of topotactic decomposition of $LaReN_3$ complicate the determination of a definite structure model as superstructure reflections are on the level of the noise. A tentative refinement of o-$LaReN_2$ in the superstructure model of o-$LnReN_2$ (Ln = Pr, Nd) was tried to obtain the $z$-position of the Re atoms. Results of the refinement are shown in Fig. S8, while refinement data are in Table S8. The quality of fit improves with respect to the undistorted model ($R_{Bragg}$ = 4.7 vs $R_{Bragg}$ = 9.4) and a Re–Re short interatomic distance of $d$ = 3.256(8) Å is indicated.

**Table S8:** Crystallographic data of the powder X-ray Rietveld refinement of o-$LaReN_2$.

| **Crystal data** | |
|---|---|
| Formula | o-$LaReN_2$ |
| Formula mass, g·mol$^{-1}$ | 355.14 |
| Crystal system | orthorhombic |
| Space group | *Cmmm* (no. 65) |
| Cell parameters, Å | $a$ = 7.950(3) |
| | $b$ = 7.1244(4) |
| | $c$ = 3.976(1) |
| Cell volume, Å$^3$ | 225.2(1) |
| Z | 4 |
| F(000) | 584 |
| Calc. density, g·cm$^{-3}$ | 10.415 |
| **Data collection** | |
| Diffractometer | Stoe & Cie StadiP |
| Radiation | Mo-$K_{\alpha 1}$ |
| Temperature, K | 298 |
| **Refinement** | |
| GOF | 0.012 |
| $R_p$, $R_{wp}$, $R_{exp}$ | 0.093, 0.123, 0.10 |
| $R_{Bragg}$ | 0.047 |

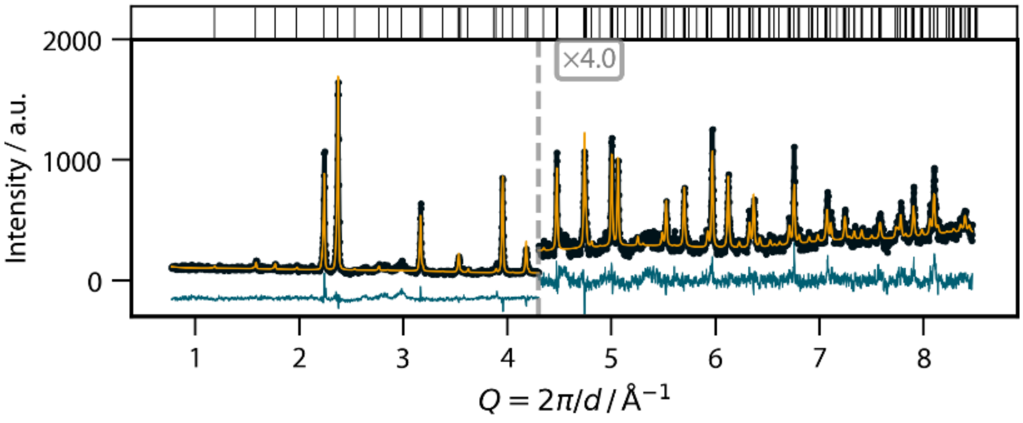


**Figure S7:** Rietveld refinements of powder X-ray diffraction data collected on $LaReN_2$. Refinement in orange, data as black circles, difference as blue line. Bragg positions are indicated as vertical lines.

## Additional information on DFT-calculations of $NdReN_2$ and t-/o-$LaReN_2$

### Nd–Nd interactions

The antiferromagnetic ground state revealed in magnetization and magnetic PND measurements (Fig. 2 and 3) is reflected in the symmetrical spin-polarized DOS curves (Figure 4). These reveal that the states close to the Fermi level stem to from the N-2p and Re-5d orbitals a large extent, accompanied by minor contributions from the Nd-5d states. The bands corresponding to the Nd-4f states are located at around −5 and 2 eV and exhibit modest dispersions. The Nd-4f states are therefore mostly localized and a contribution to potential metal−metal bonds is hindered. The computed magnetic moment of $\mu_{\mathrm{eff}} = 3.02\ \mu_{\mathrm{B}}$ is slightly reduced, in line with the results of the PND data. This may be rationalized along the known hybridization of Nd-4f states with the Nd-5d orbitals, leading to a certain delocalization and bonding contributions from the 4f electrons.[30]

The bonding analyses for the short Nd−Nd contacts perpendicular to the $ReN_{4/2}$ sheets with values of $ICOBI_\alpha = 0.091$ and $-IpCOHP_\alpha = 0.86$ eV make bonding interactions evident. These interaction should mediate direct exchange interactions between the neodymium atoms, which is reflected in the ferromagnetic alignment of magnetic moment vectors stacked perpendicular to the $ReN_{4/2}$ sheets (Figure 3a), which is reminiscent of the one-dimensional ferromagnetic Heisenberg chain.[31] Within the Nd-sheets, shorter Nd−Nd separations (3.800(1) Å) correspond to modest, but evident $ICOBI_\alpha$ (0.088) and –Ip-$COHP_\alpha$ (0.56 eV) values and could also mediate direct exchange interactions. Values of the remaining Nd−Nd contacts within the layers ($d_{\mathrm{Nd–Nd}} > 3.90$ Å: $ICOBI_\alpha < 0.055$, $-IpCOHP_\alpha < 0.29$ eV) point to a less bonding character relative to the aforementioned shorter Nd−Nd bonds. The value of the experimental critical exponent β, located between the values of the 3D and 2D Ising models, reflect the multiple sources of exchange interactions.

### Comparison of t-$LaReN_2$ and o-$LaReN_2$

A comparison of the total energies for tetragonal and orthorhombic $LaReN_2$ models (Figure S9) reveals that the net cohesive energy corresponding to a hypothetical orthorhombic structure is 9.5 meV lower than that of the recently reported tetragonal structure model. Accordingly, the formation of the orthorhombic structure model should be preferred. However, the energy difference between these structure models is rather small so that the formation of the tetragonal structure model could be a consequence of a non-equilibrium condensation into the solid state. This outcome indicates that an orthorhombic $LaReN_2$ should exist. In light of the poor crystal quality of the topotactically reduced samples, further explorations using different reaction conditions could be helpful to gain further access to the orthorhombic modification.

To analyse why the energy difference between the two structures is quite small, a bonding analysis for both $LaReN_2$ models is performed. A population analysis shows that an ionic scenario as suggested by the formula $(La^{3+})(Re^{3+})(N^{3-})_2$ is not present in both models. The nature of the La−N and Re−N interactions is best described as polar-covalent, while metallic bonding is evident for La−La and La−Re interactions. An additional comparison of both $LaReN_2$ structure models brings to light that the coordination environment of every site in the orthorhombic model is distorted relative those in the tetragonal $LaReN_2$. That distortion of the coordination polyhedra does not only translate into a reduction of local symmetry, but also leads to a broader spectrum of distances in the orthorhombic than in the tetragonal structure model. Under consideration of Pauling´s fifth rule,[32] one could assume that the ordered tetragonal model should be preferred rather than the orthorhombic species; however, the bonding analysis clearly ruled out that just ionic bonds as considered in the framework of Pauling´s rules are present. The broader spectrum of bonds in the orthorhombic $LaReN_2$ means that stronger as well as weaker bonds are formed relative to those in the tetragonal $LaReN_2$ − a scenario that can be easily recognized from a

comparison of the respective ICOBI/bond values. When evaluating the cumulative –IpCOHP/f.u. values, there is an increase in energy from the tetragonal to the orthorhombic $LaReN_2$.

In summary, the outcome of the bonding analysis shows that the formation of the orthorhombic model is preferred in order to enhance the bonding situation. The modest gain in energy though means that the tetragonal model can also be observed for $LaReN_2$.

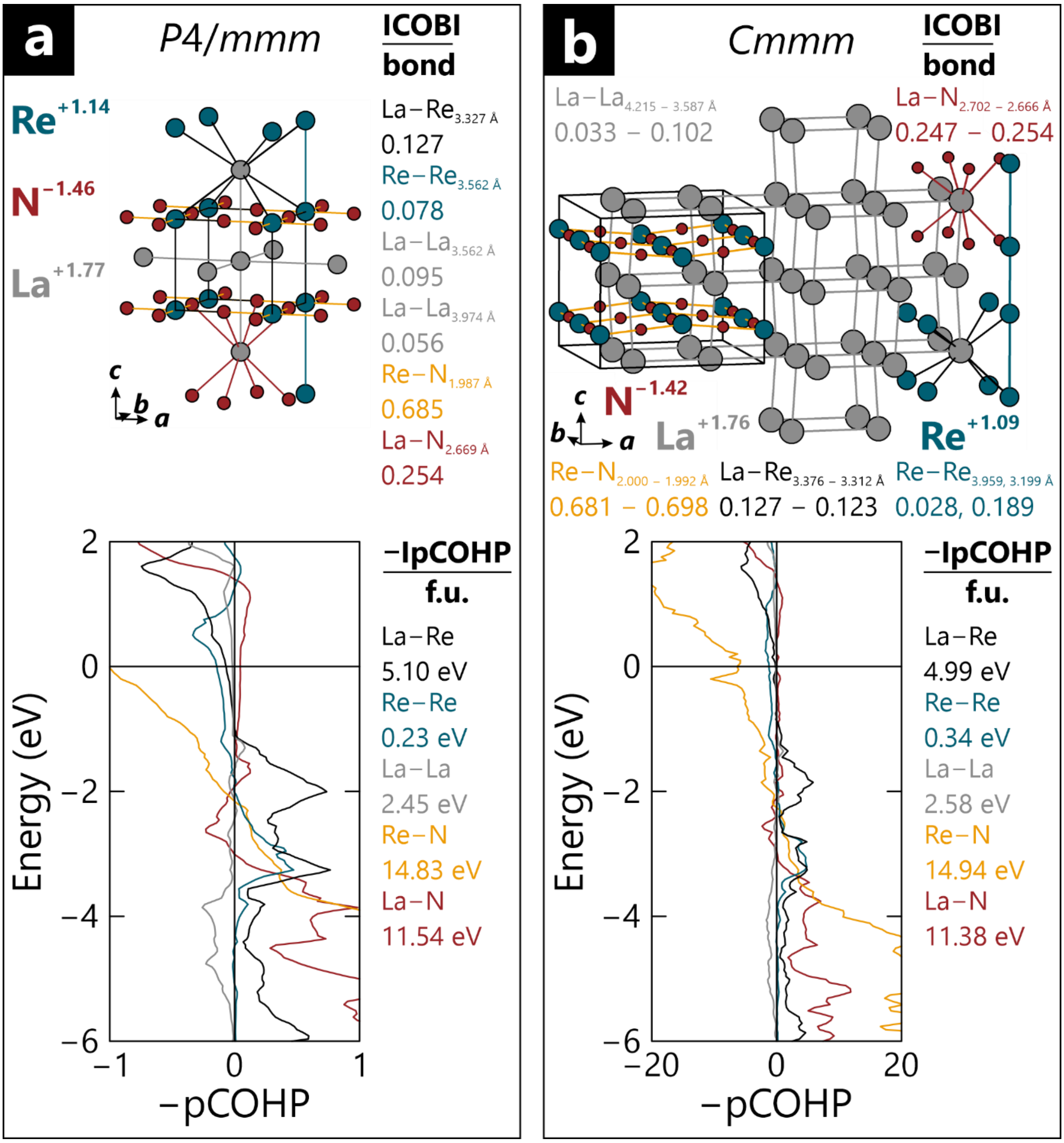


**Figure S8:** averaged Bader charges (in units of the elementary charge e), cumulative −pCOHP/formula unit (f.u.), ICOBI/bond values, −pCOHP diagrams of (a) the recently reported t-$LaReN_2$ and (b) a hypothetical o-$LaReN_2$, whose structure model is constructed based on that of the herein reported $NdReN_2$.

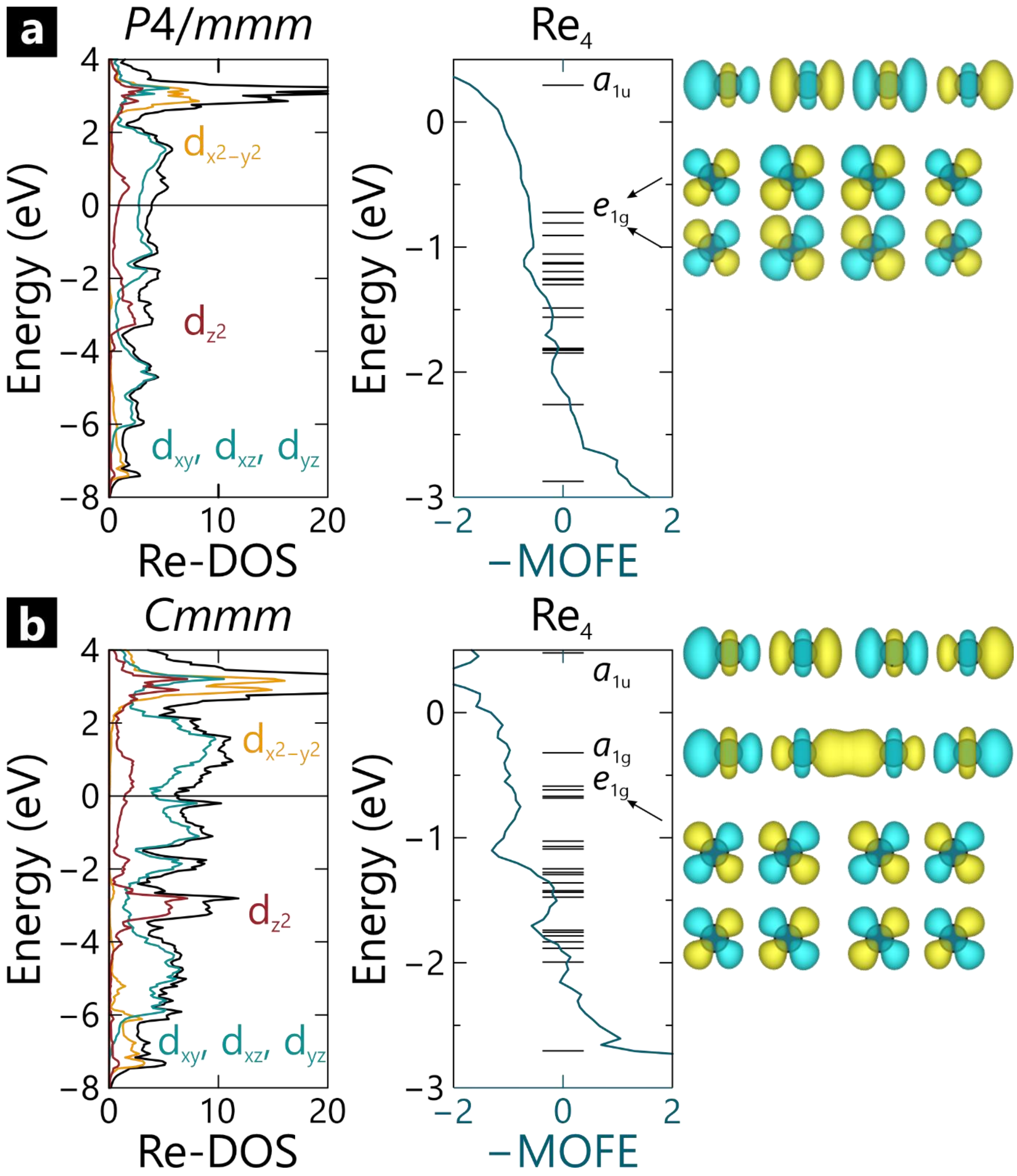


**Figure S9:** Re-DOS and MOFE of a $Re_4$ unit for (a) the recently reported t-$LaReN_2$ and (b) a hypothetical o-$LaReN_2$, whose structure model is constructed based on that of the herein reported $NdReN_2$.

To explore the nature of the distortion in the chains in more detail, the MO diagrams for $Re_4$ and $ReN_4$ units in t- as well as o-$LaReN_2$ are further analyzed. Both cases of the ReN4 units (Fig. S11) show a bonding scheme as expected for a square planar complex. The Re-6s and $5d_{z2}$ atomic orbitals combine with the nitrogen atomic orbitals to establish low-lying molecular orbitals corresponding to $a_{1g}$ and $a_1$ sets in the tetragonal and orthorhombic models, respectively. The decrease in symmetry from the tetragonal structure model to the orthorhombic one leads to the change in orbital symmetry. The states related to the Re-$5d_{xy}$ orbitals are slightly lower in energy than the bands arising from the Re-$5d_{yz}$ and Re-$5d_{xz}$ states. The HOMOs of the $ReN_4$ units correspond to an $a_{1g}$ orbital in t-$LaReN_2$ and to an $a_1$ orbital in o-$LaReN_2$ and exhibit contributions arising from the Re-6s and -$5d_{z2}$ orbitals. While a mixing occurring

between the transition metal-s and -$d_{z2}$ orbitals is typically encountered for transition metal-centered complexes showing a Jahn−Teller-type distortion, the gain in energy that is related to that the distortion of the $ReN_4$ units is rather modest. An integration of the molecular orbital formation energy (MOFE) curves reveals that the –IMOFE value related to the orthorhombic model is just 0.06 eV larger than that determined for the tetragonal one. However, the –IMOFE value related to the $Re_4$ units in the orthorhombic structure (1.95 eV) is more than twice as large as that of the $Re_4$ units in the tetragonal structure model (0.84 eV). A closer analysis of the Re-based MO and DOS diagrams shows that the bands corresponding to the Re-5$d_{z2}$ states split up, as the linear $Re_4$ chains distort from t-$LaReN_2$ to o-$LaReN_2$. As a consequence, there is a noticeable maximum of the DOS which corresponds to the Re-5$d_{z2}$ states in o-$LaReN_2$ (at ~−3 eV, Figure S10), while such a maximum is not evident in the Re-5$d_{z2}$ DOS in t-$LaReN_2$. The HOMO of the $Re_4$ units in o-$LaReN_2$ originates from the Re-5$d_{z2}$ states, which are not significant in the HOMOs of the $Re_4$ units in t-$LaReN_2$. Accordingly, the structural distortions of the chains correlates with an energetically favorable splitting up of the Re-5$d_{z2}$ bands thereby approaching the Peierls theorem.

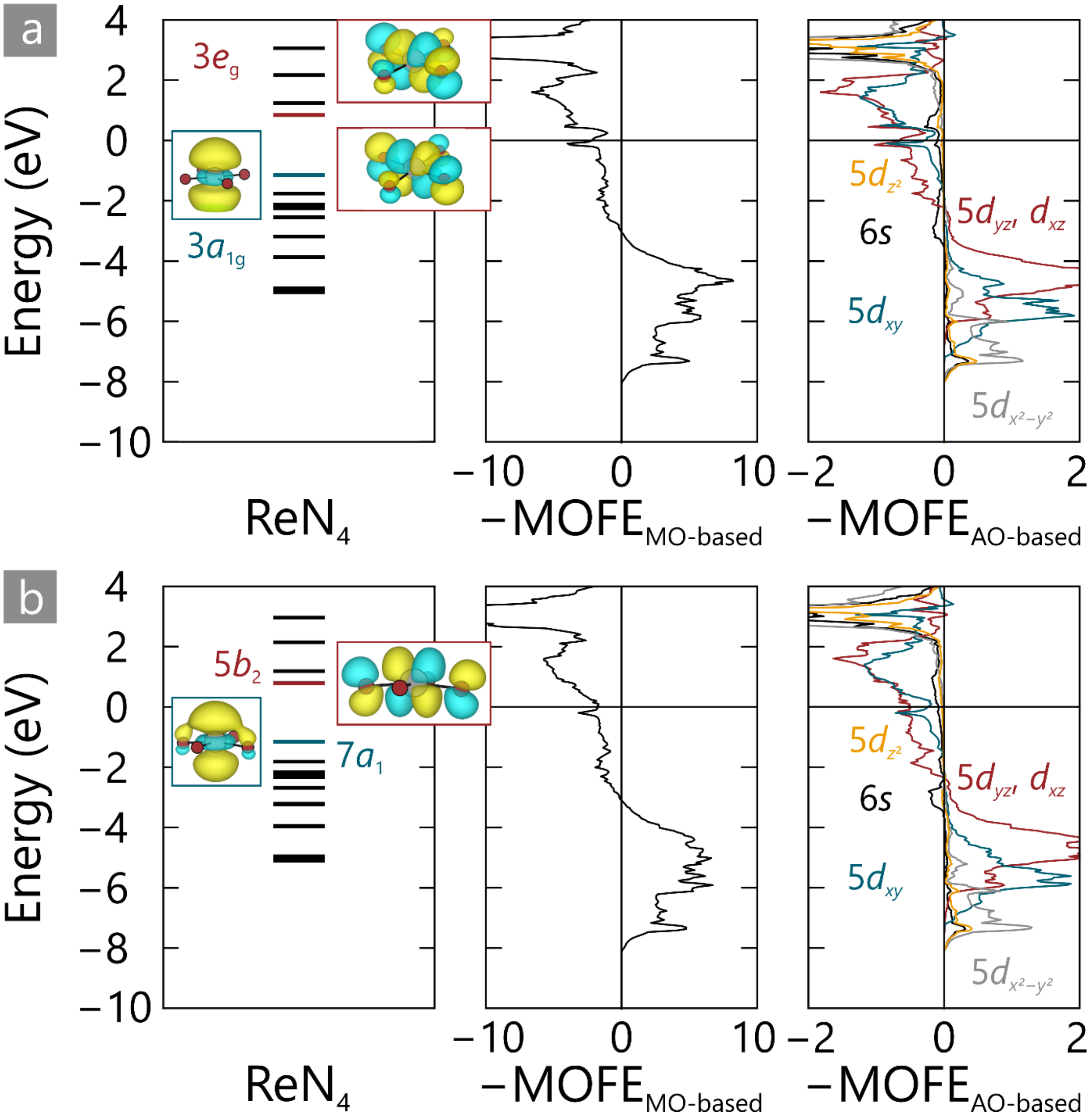


**Figure S10:** MO diagram, MO-based and Re-based MOFE of a $ReN_4$ unit for (a) the recently reported t-$LaReN_2$ and (b) a hypothetical o-$LaReN_2$.

# High Temperature Powder X-ray Diffraction

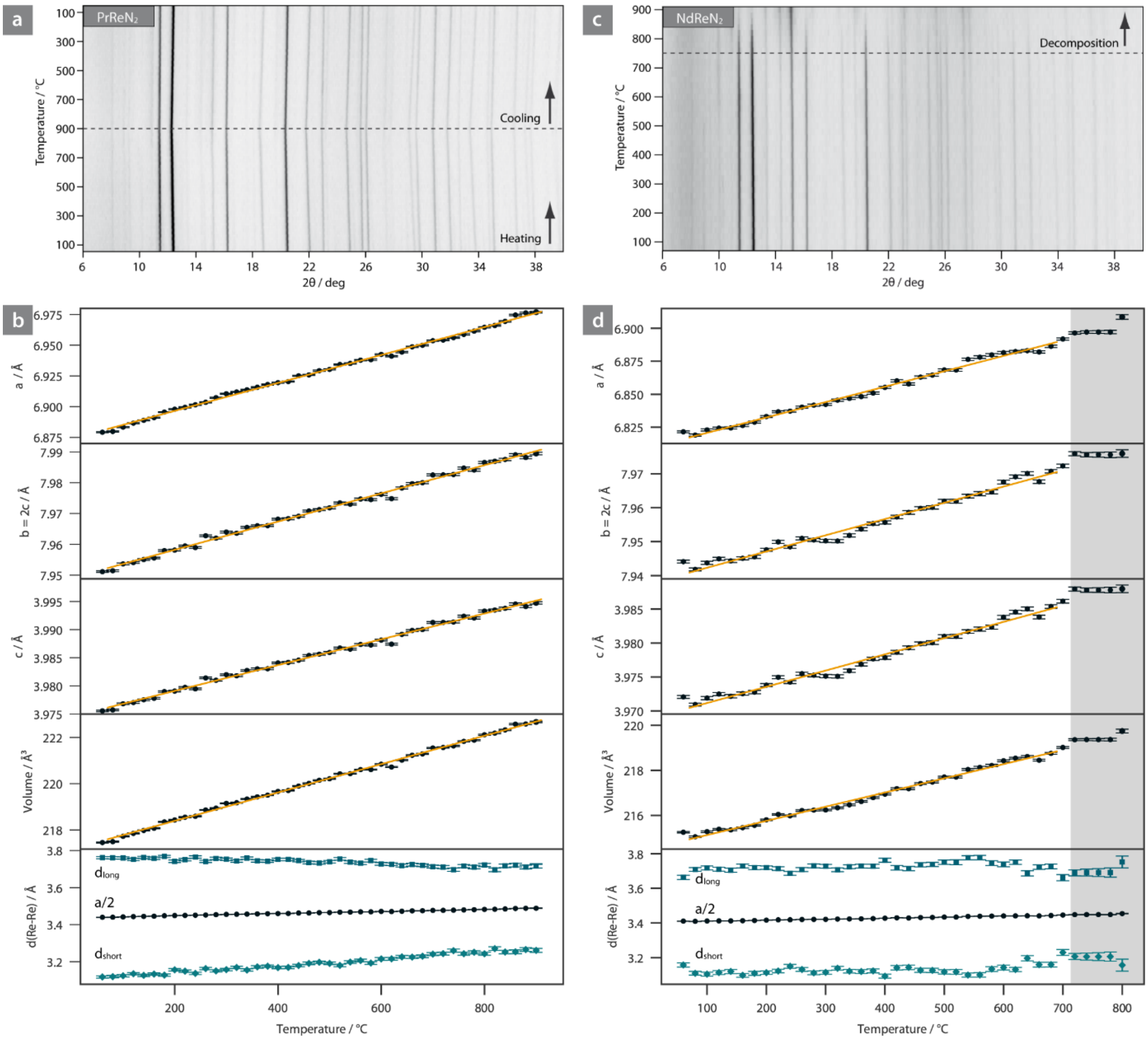


**Figure S11:** Temperature-dependent powder X-ray diffraction of $LnReN_2$. Measured data are shown for $PrReN_2$ in **a** and for $NdReN_2$ in **c**. The switch from heating to cooling in $PrReN_2$ and the onset of decomposition at around 750 °C of $NdReN_2$ are indicated as horizontal dashed lines. Results of Rietveld-analysis are shown in for $PrReN_2$ in **b** and for $NdReN_2$ in **d** with lattice parameters displayed as black circles and errors as horizontal bars. The lattice parameters *b* were constrained to be equal to 2*c* for the refinement as the resolution of the diffractometer could not resolve the superstructure reflections. Orange lines are fits of linear thermal expansion. The evolution of the Re–Re interatomic distances (short and long contacts) are displayed in the lowest panel together with lattice parameter *a*/2 as black circles.

## References


[1] A. A. Coelho, *J. Appl. Cryst.* **2018**, *51*, 210-218.
[2] K. Momma, F. Izumi, *J. Appl. Crystallogr.* **2011**, *44*, 1272-1276.
[3] S. D. Kloß, M. L. Weidemann, J. P. Attfield, *Angew. Chem. Int. Ed.* **2021**, *60*, 22260-22264.
[4] P. E. Blöchl, *Phys. Rev. B: Condens. Matter Mater. Phys.* **1994**, *50*, 17953-17979.
[5] G. Kresse, J. Hafner, *Phys. Rev. B: Condens. Matter Mater. Phys.* **1993**, *47*, 558-561.
[6] G. Kresse, J. Furthmüller, *Comput. Mater. Sci.* **1996**, *6*, 15-50.
[7] G. Kresse, J. Furthmüller, *Phys. Rev. B: Condens. Matter Mater. Phys.* **1996**, *54*, 11169-11186.
[8] G. Kresse, D. Joubert, *Phys. Rev. B: Condens. Matter Mater. Phys.* **1999**, *59*, 1758-1775.
[9] G. Kresse, M. Marsman, J. Furthmüller, *Vienna Ab-initio Simulation Package VASP: the Guide*, Computational Materials Physics, Faculty of Physics, Universität Wien, Vienna, Austria **2014**.
[10] J. P. Perdew, K. Burke, M. Ernzerhof, *Phys. Rev. Lett.* **1996**, *77*, 3865-3868.
[11] J. K. Lang, Y. Baer, P. A. Cox, *J. Phys. F: Met. Phys.* **1981**, *11*, 121-138.
[12] R. Dronskowski, P. E. Blöchl, *J. Phys. Chem.* **1993**, *97*, 8617-8624.
[13] V. L. Deringer, A. L. Tchougréeff, R. Dronskowski, *J. Phys. Chem. A* **2011**, *115*, 5461-5466.
[14] S. Steinberg, R. Dronskowski, *Crystals* **2018**, *8*, 225.
[15] P. C. Müller, C. Ertural, J. Hempelmann, R. Dronskowski, *J. Phys. Chem. C* **2021**, *125*, 7959-7970.
[16] C. Ertural, S. Steinberg, R. Dronskowski, *RSC Adv.* **2019**, *9*, 29821-29830.
[17] R. F. W. Bader, *Chem. Rev.* **1991**, *91*, 893-928.
[18] G. Henkelman, A. Arnoldson, H. Jónsson, *Comput. Mater.* **2006**, *36*, 354-360.
[19] E. Sanville, S. D. Kenny, R. Smith, G. Henkelman, *J. Comput. Chem.* **2007**, *28*, 899-908.
[20] W. Tang, E. Sanville, G. Henkelman, *J. Phys.: Condens. Matter.* **2009**, *21*, 084204.
[21] M. Yu, D. R. Trinkle, *J. Chem. Phys.* **2011**, *134*, 064111.
[22] S. Maintz, V. L. Deringer, A. L. Tchougréff, R. Dronskowski, *J. Comput. Chem.* **2013**, *34*, 2557-2567.
[23] S. Maintz, V. L. Deringer, A. L. Tchougréff, R. Dronskowski, *J. Comput. Chem.* **2016**, *37*, 1030-1035.
[24] R. Nelson, C. Ertural, J. George, V. L. Deringer, G. Hautier, R. Dronskowski, *J. Comput. Chem.* **2020**, *41*, 1931-1940.
[25] B. Eck, *wxDragon 2.2.3*, RWTH Aachen University, Aachen, Germany, RWTH Aachen University, Aachen, Germany, **2020**.
[26] K. Brandenburg, *DIAMOND (Version 4.6.3), Crystal and Molecular Structure Visualization*, K. Brandenburg & H. Putz GbR, Bonn, Germany, **2020**.
[27] P. C. Muller, N. Schmit, L. Sann, S. Steinberg, R. Dronskowski, *Inorg. Chem.* **2024**, *63*, 20161-20172.
[28] O. Reckeweg, H. J. Meyer, *Z. Anorg. Allg. Chem.* **1999**, *625*, 866-874.
[29] M. Woike, W. Jeitschko, *Inorg. Chem.* **1995**, *34*, 5105-5108
[30] K. A. Gschneidner, *J. Alloys Comp.* **1993**, *192*, 1-10.
[31] W. Heisenberg, *Z. Phys.* **1928**, *49*, 619-636.
[32] L. Pauling, *J. Am. Chem. Soc.* **1929**, *51*, 1010-1026.
